# Identification of a novel prognostic marker ADGRG6 in pancreatic adenocarcinoma: multi-omics analysis and experimental validation

**DOI:** 10.3389/fimmu.2025.1530789

**Published:** 2025-03-27

**Authors:** Han Wu, Jin Shang, Yuanyan Bao, Huajie Liu, Haoran Zhang, Yaosheng Xiao, Yangtaobo Li, Zhaozhang Huang, Xiaolei Cheng, Zixuan Ma, Wenqing Zhang, Pingli Mo, Daxuan Wang, Mingqing Zhang, Yanyan Zhan

**Affiliations:** ^1^ Cancer Research Center, School of Medicine, Xiamen University, Xiamen, Fujian, China; ^2^ Department of Gastroenterology, The 909th Hospital, School of Medicine, Xiamen University, Zhangzhou, Fujian, China; ^3^ Department of Infectious Disease, Xiang’an Hospital Affiliated to Xiamen University, Xiamen, Fujian, China; ^4^ State Key Laboratory of Cellular Stress Biology, Innovation Center for Cell Biology, School of Life Science, Xiamen University, Xiamen, Fujian, China; ^5^ Provincial College of Clinical Medicine, Fujian Provincial Hospital, Fuzhou, Fujian, China

**Keywords:** pancreatic cancer, ADGRG6, mutated p53, prognosis marker, membrane receptors

## Abstract

**Background:**

Pancreatic adenocarcinoma (PAAD) ranks among the most lethal malignancies worldwide. Current treatment options have limited efficacy, underscoring the need for new therapeutic targets.

**Methods:**

This study employed a multi-omics analytical framework to delve into the expression profiles and prognostic implications of ADGRG6 within the pan-cancer dataset of The Cancer Genome Atlas (TCGA) database, highlighting the prognostic value and potential carcinogenic role of ADGRG6 in PAAD, which was further validated using data from multiple PAAD cohorts in the Gene Expression Omnibus (GEO) database. To assess the role of ADGRG6 in the tumor microenvironment of PAAD, we evaluated immune infiltration using CIBERSORT, ssGSEA, xCell and Tracking Tumor Immunophenotype (TIP), and utilized single-cell sequencing data to explore cell-cell interactions. Further cellular and animal experiments, such as colony formation assay, transwell assay, western blot, real-time PCR, and tumor xenograft experiments, were used to investigate the effect of ADGRG6 on the proliferation, metastatic potential and immune marker expression of PAAD and the underlying mechanisms.

**Results:**

ADGRG6 emerged as a potential prognostic biomarker and a therapeutic target for PAAD, which was further corroborated by data extracted from multiple PAAD cohorts archived in the GEO database. Single-cell sequencing and immune infiltration analyses predicted the positive correlation of ADGRG6 with the infiltration of immune cells and with the interaction between malignant cells and fibroblasts/macrophages within the PAAD microenvironment. *In vitro* cell assays demonstrated that ADGRG6 promoted the proliferation, metastatic potential and immune marker expression of PAAD cells by increasing protein level of mutated p53 (mutp53), which activated a spectrum of gain-of-functions to promote cancer progression via the EGFR, AMPK and NF-κB signaling cascades. Furthermore, subcutaneous xenograft experiments in mice demonstrated that ADGRG6 knockdown substantially suppressed the growth of engrafted PAAD tumors.

**Conclusions:**

ADGRG6 may serve as a novel prognostic marker and a therapeutic targets for PAAD, playing a crucial role in the proliferation, metastasis, and immune marker regulation of PAAD through elevating protein level of mutated p53.

## 1 Introduction

Pancreatic adenocarcinoma (PAAD) is among the deadliest cancers globally, characterized by a dismal prognosis and a low 5-year survival rate (1). The disease develops insidiously and is frequently diagnosed at advanced stages, compounded by a complex tumor microenvironment that complicates the development of effective treatment strategies (2–4). While surgery combined with chemotherapy remains the primary treatment approach, its effectiveness is limited (5, 6). With advancements in precision medicine, molecular-targeted therapies have emerged as a prominent treatment modality for various cancers. However, these therapies currently exhibit poor clinical outcomes for PAAD (7, 8). Consequently, there is an urgent need to identify new molecular targets for more effective treatments of PAAD.

Multi-omics technologies are advancing rapidly, significantly enhancing our understanding of PAAD, which exhibits substantial molecular heterogeneity. Whole exome sequencing has identified four common classes of somatic mutations occurring in the oncogene KRAS and the tumor suppressor genes TP53, CDKN2A, and SMAD4 (9, 10). Transcriptome and single-cell sequencing have introduced various molecular typing methods that cater to the needs of personalized treatment (11–14). Additionally, single-cell sequencing and spatial transcriptomics have greatly facilitated the exploration of the unique microenvironment of PAAD, thereby expanding treatment options (15, 16).

ADGRG6, also known as GPR126, is a member of the adhesion G protein-coupled receptor family, characterized by its seven-transmembrane structure and its role in regulating cell adhesion and signaling (17–19). Current research on ADGRG6 primarily focuses on its neurological functions. ADGRG6 promotes myelination through Gas/cAMP/PKA signaling pathway, and is crucial for peripheral nerve regeneration and Schwann cell development (20–23). Abnormal expression and potential role of ADGRG6 were recently implicated in several cancers, including colon, breast, and bladder cancers (24–26). However, there is a notable gap in comprehensive analysis of this gene across pan-cancer studies, and its expression and role in PAAD remains unclear.

In this study, we examined the expression patterns, epigenetics, and prognostic value of ADGRG6 in The Cancer Genome Atlas (TCGA) pan-cancer cohort using multi-omics approaches. The analyses strikingly suggested that ADGRG6 had potential as both a prognostic marker and a therapeutic target for PAAD, which was further supported by data from multiple PAAD cohorts from the Gene Expression Omnibus (GEO) database. Furthermore, single-cell sequencing and immune infiltration analyses predicted the possible role of ADGRG6 in the PAAD microenvironment. Additionally, our series of cell experiments demonstrated that ADGRG6 knockdown significantly inhibited while ADGRG6 overexpression enhanced the growth and metastasis of PAAD cells by regulating the expression level of mutated p53 (mutp53) protein, which exerted various gain-of-function (GOF) activities through EGFR and NF-κB signaling pathways. Subcutaneous transplantation experiments in nude mice indicated that ADGRG6 knockdown substantially suppressed growth of xenografted tumor of PAAD cells. Therefore, these findings illuminate the role of ADGRG6 in PAAD development and offer potential targets for molecular targeted therapies of PAAD.

## 2 Materials and methods

### 2.1 Dataset resources

The TCGA and the Genotype-Tissue Expression (GTEx) pan-cancer datasets, which include normalized expression profiles, clinical information, somatic mutation data, and survival data, were downloaded from UCSC (https://xenabrowser.net/datapages/), cBioPortal (https://www.cbioportal.org/), and the R package “TCGAbiolinks”. Protein levels across various cancer types were assessed using cProSite (https://cprosite.ccr.cancer.gov/). The relationships between ADGRG6 and clinical characteristics, as well as survival times, were analyzed using data from GEO (https://www.ncbi.nlm.nih.gov/geo/), which includes datasets GSE71729, GSE15471, GSE183795, GSE62452, GSE28735, GSE57495, GSE21501, and GSE85916. PAAD cohort data related to ICGC-AU was retrieved from International Cancer Genome Consortium (ICGC, https://dcc.icgc.org/). Kaplan-Meier survival curves were generated using the Kaplan-Meier Plotter (KM-plot, https://kmplot.com/analysis/index.php?p=service) to evaluate the potential of ADGRG6 as a survival marker for pancreatic cancer. Additionally, PAAD single-cell datasets were downloaded from GEO (https://www.ncbi.nlm.nih.gov/geo/).

### 2.2 Prognostic analysis

For the TCGA pan-cancer cohort, Cox regression analysis on the four survival indicators—overall survival (OS), progression-free interval (PFI), disease-specific survival (DSS), and disease-free interval (DFI)—was conducted using the R package “survival”. The R packages “survival” and “survminer” were employed to determine the optimal cutoff values and generate Kaplan-Meier curves to assess the prognostic value of ADGRG6.

### 2.3 Gene functional enrichment and immunology analysis

Gene functional enrichment analysis was performed using collections downloaded from the Molecular Signatures Database (MSigDB, https://www.gsea-msigdb.org/gsea/msigdb) and the R package “clusterProfiler”. Gene set scoring was conducted with the R package “GSVA”. To explore the relationship between ADGRG6 expression and tumor immune infiltration, we utilized the “cibersort” algorithm (via the R package “CIBERSORT”), “ssGSEA” (via the R package “GSVA”), and “xCell” (via the R package “xCell”). Additionally, the Tracking Tumor Immunophenotype (TIP, http://biocc.hrbmu.edu.cn/TIP/) tool was employed to predict potential correlations between ADGRG6 expression and the tumor immune cycle.

### 2.4 Single-cell sequencing analysis

Single-cell sequencing data were downloaded from GEO and processed using the Seurat R package. For quality control, cells expressing fewer than 200 genes or more than 20% mitochondrial reads were removed. The “doubletFinder” function from the R package “doubletFinder” was employed to identify and exclude potential doublets in each sample. The filtered data were then scaled and normalized prior to advanced analysis. Batch effects between samples were corrected using the R package “Harmony”. Cell clustering and dimensionality reduction were performed with the “FindClusters” and “RunUMAP” functions. To annotate cell types, “FindAllMarkers” was used to identify specific genes within each cell cluster. Copy number variation (CNV) was assessed with the R package “CopyKAT” to differentiate malignant cells from normal cells. Cell-cell interactions among different cell types were evaluated using CellChat with the default parameters of the database, focusing exclusively on receptor-ligand interactions as annotated in the database.

### 2.5 Antibodies and reagents

Anti-ADGRG6 (Cat. #TA315653, OriGene), anti-p53 (Cat. #3H2821, Santa Cruz Biotechnology), β-actin (Cat. #A2066, Sigma-Aldrich), anti-N-cadherin (Cat. #393933, Santa Cruz Biotechnology), anti-PCNA (Cat. #2586, Cell Signaling Technology), anti-EGFR (Cat. #4267, Cell Signaling Technology), anti-p-EGFR (Cat. #2234, Cell Signaling Technology), anti-p65 (Cat. #8242, Cell Signaling Technology), anti-AMPK (Cat. #2532, Cell Signaling Technology), anti-p-AMPK (Cat. #2531, Cell Signaling Technology) antibodies were used. HRP-conjugated affinipure donkey anti-rabbit IgG(H+L) (Cat.SA00001-9) and goat anti-mouse IgG(H+L) (Cat. SA00001-1) antibodies were purchased from Proteintech Group (Wuhan, China).

Transwell chamber apparatus (Cat. #3422) and Matrigel (Cat. #354234) were purchased from Corning (New York, NY, USA). MTT powder were purchased from Sigma (Louis, MO, USA).

### 2.6 Cell culture

Human pancreatic cancer cell lines (AsPC-1, CFPAC-1, MIAPaca-2, BxPC-3, and PANC-1) and HEK293T cells were purchased from the Cell Bank of the Chinese Academy of Sciences, Type Culture Collection, Shanghai, China. Human normal pancreatic ductal cells (hTERT-HPNE) were acquired from the American Type Culture Collection (ATCC, Manassas, VA, USA). All cell lines were cultured in media supplemented with 10% fetal bovine serum (FBS, Sigma) at 37 °C in a 5% CO_2_ incubator. AsPC-1 cells were grown in RPMI 1640 medium (Gibco), CFPAC-1 cells in Iscove’s Modified Dulbecco’s Medium (IMDM, Gibco), and HEK293T, BxPC-3, PANC-1, and MIAPaca-2 cells in Dulbecco’s Modified Eagle’s Medium (DMEM, Gibco). The complete medium consisted of base culture media, 10% fetal bovine serum, 100 U·mL^-1^ penicillin, and 100 μg·mL^-1^ streptomycin (Life Technologies, Carlsbad, CA, USA).

### 2.7 Construction of stable knockdown and overexpression cell lines by lentivirus

ShRNA oligos targeting ADGRG6 were annealed and subcloned into the pLKO.1-puro plasmid. For overexpression, the ADGRG6 or mutated p53 gene was subcloned into the pLVX-3Flag-Puro plasmid. HEK293T cells were transfected with PEI40000 and packaging plasmids (pMDLg-pRRE, pRSV-REV, and pCMV-VSV-G) to produce lentivirus. After 48 hours, the lentiviral supernatant was collected, centrifuged at 1600 rpm for 10 minutes, and filtered through 0.45 μm filters (Millipore, Billerica, MA, USA). The filtered lentiviral supernatant was added to the culture medium of the human pancreatic cancer cell lines. Following a 48-hour infection period, puromycin (2 μg·mL^-^¹) was used to select for stable knockdown/overexpression cell lines.

The shRNA sequences used were as follows:

shCtrl: CAACAAGATGAAGAGCACCAA;
shADGRG6-1: ACAGAAACAATCGCCAAATAT;
shADGRG6-2: TTCAAACAGCAGGAGATAATT;
shP53-1:TATCCGAGTGGAAGGAAATTT;
ShP53-2:ACCACTGGATGGAGAATATTT.

### 2.8 Real-time PCR

Total RNA was extracted from cells using Trizol (Takara, Dalian, China) and reverse-transcribed into cDNA using a Synthesis SuperMix kit (Yeasen, Shanghai, China). Real-time PCR was conducted using the qPCR SYBR Green Master Mix kit (Yeasen, Shanghai, China) according to the manufacturer’s instructions. The *ACTB* gene served as an internal control.

The primers used were as follows:

*ADGRG6*: F: 5′-ACAGAGCAAGGTGGCAGAATGG-3′;
     R: 5′-TTGTCCTCTCCAGCACTCAGGT-3′;
*CDH1*:   F: 5′-GCCTCCTGAAAAGAGAGTGGAAG-3′;
     R: 5′-TGGCAGTGTCTCTCCAAATCCG-3′;
*CDH2*:   F: 5′-CCTCCAGAGTTTACTGCCATGAC-3′;
     R: 5′-GTAGGATCTCCGCCACTGATTC-3′;
*Snail1*:   F: 5′-TGCCCTCAAGATGCACATCCGA-3′;
     R: 5′-GGGACAGGAGAAGGGCTTCTC-3′;
*Twist*:    F; 5′-GTCCGCAGTCTTACGAGGAG-3′;
     R: 5′-GCTTGAGGGTCTGAATCTTGCT-3′;
*Vimentin*: F;5′-AGGCAAAGCAGGAGTCCACTGA-3′;
     R: 5′-ATCTGGCGTTCCAGGGACTCAT-3′;
ZEB1:    F: 5′-GGCATACACCTACTCAACTACGG-3′;
     R: 5′-TGGGCGGTGTAGAATCAGAGTC-3′;
ACTB:   F: 5′-GTTGCTATCCAGGCTGTGCT-3′;
     R: 5′-AGGTAGTCAGTCAGGTCCCG-3′;
CD274:  F: 5′-TGGCATTTGCTGAACGCATTT-3′;
     R: 5′-TGCAGCCAGGTCTAATTGTTTT-3′;
SIGLEC15: F: 5′-CGCGGATCGTCAACATCTC-3′;
     R: 5′-GTTCGGCGGTCACTAGGTG-3′;
CD44:   F: 5′-CTGCCGCTTTGCAGGTGTA-3′;
     R: 5′-CATTGTGGGCAAGGTGCTATT-3′;
LGALS9:  F: 5′-GGACGGACTTCAGATCACTGT-3′;
     R: 5′-CCATCTTCAAACCGAGGGTTG-3′;
ITGAV: F: 5′-ATCTGTGAGGTCGAAACAGGA-3′;
     R: 5′-TGGAGCATACTCAACAGTCTTTG-3′;
ITGB5:  F: 5′-TCTCGGTGTGATCTGAGGG-3′;
     R: 5′-TGGCGAACCTGTAGCTGGA-3′;
ITGB6:  F: 5′-TCCATCTGGAGTTGGCGAAAG-3′;
     R: 5′-TCTGTCTGCCTACACTGAGAG-3′;

### 2.9 Western blot

Cells were lysed using ELB lysis buffer (150 mM NaCl, 100 mM NaF, 25 mM Tris-HCl [pH 7.6], 1% Nonidet P-40 [NP-40], 1 mM PMSF, and protease inhibitors) for 10 minutes on ice. The lysate was collected with a scraper, subjected to ultrasonic treatment, and then centrifuged at 13,000 rpm for 15 minutes at 4°C. The supernatant was collected and protein concentration was determined. Proteins were separated by SDS-PAGE, transferred to a PVDF membrane, and blocked with 5% skim milk. The membrane was incubated overnight at 4°C with specific primary antibodies. The following day, it was incubated with appropriate fluorescent secondary antibodies. Immunoreactive bands were detected using an Enhanced Chemiluminescence (ECL) system (Bio-Rad, Hercules, CA, USA).

### 2.10 Transwell assay

The Transwell chamber apparatus was used for the assay. Before the invasion experiment, a 10% matrix gel was prepared and 50 μL of this gel was added to the upper chamber, allowing the gel to solidify. For both the migration and the invasion assays, a specified number of cells were seeded in the upper chamber (cells plated in each Transwell chamber: CFPAC-1: 6×10^4^, Panc-1: 5×10^4^, MIAPaca-2: 10×10^4^). After 24 hours, non-invading cells in the upper chamber were removed with cotton swabs. The invading cells on the lower side of the membrane were fixed with 4% paraformaldehyde for 5-10 minutes, dried, and stained with 0.1% crystal violet for 5-10 minutes. Three fields of view from each well were captured for statistical analysis.

### 2.11 Cell proliferation assay

In the MTT assay, cells were seeded in a 96-well plate. After 12 hours, 20 μL of MTT solution was added to each well, and the plate was incubated for an additional 4 hours. Following incubation, the medium was replaced with 200 μL of DMSO, and the plate was shaken for 15 minutes to dissolve the formazan crystals produced by MTT. The absorbance was measured at 570 nm using a microplate reader. This procedure was repeated for 5 consecutive days (initial number of cells plated: CFPAC-1: 3000, Panc-1: 3000, MIAPaca-2: 2500).

In the colony formation assay, a small number of cells were seeded in a six-well plate. Once colonies formed (approximately 14 days), the culture medium was discarded, and the cells were fixed with 4% paraformaldehyde. The colonies were then stained with 0.1% crystal violet. Three fields of view from each well were captured for statistical analysis (initial number of cells plated: CFPAC-1: 1000, Panc-1: 1000, MIAPaca-2: 500).

### 2.12 Tumor xenograft experiments

CFPAC-1 cells (1.5 × 10^6^ per mouse) were suspended in 200 μL of base DMEM medium (without serum) and injected subcutaneously into the right flank of each nude mouse. Seven days later, when tumors became visible, measurements were taken daily, and tumor volume (Tv) was calculated using the formula: Tv = L (length) × W^2^ (width)/2. At the end of the observation period, mice were euthanized, weighed, and tumor samples were collected for Western blot analysis.

Animal experiments were conducted according to protocols approved by the Animal Care and Use Committee of Xiamen University. Female nude mice (BALB/c, 15–20 g, 5–6 weeks old) were obtained from the SLAC Laboratory Animal Center (Shanghai, China). The mice were housed and handled under specific pathogen-free conditions at the Laboratory Animal Center of Xiamen University (Xiamen, China).

### 2.13 Statistical analysis

For bioinformatics analysis, statistical computations were performed using R software (version). Specific tests such as the Wilcoxon rank-sum test and Log-rank test were applied. In biological experiments, data were presented as means ± SEM. Statistical comparisons between groups were made using Student’s t-test or one-way ANOVA, with significance determined by GraphPad Prism 8 (GraphPad Software) and Image J. A *p*-value of < 0.05 was considered statistically significant.

## 3 Result

### 3.1 ADGRG6 served as a potential biomarker for PAAD

**Figure 1:**
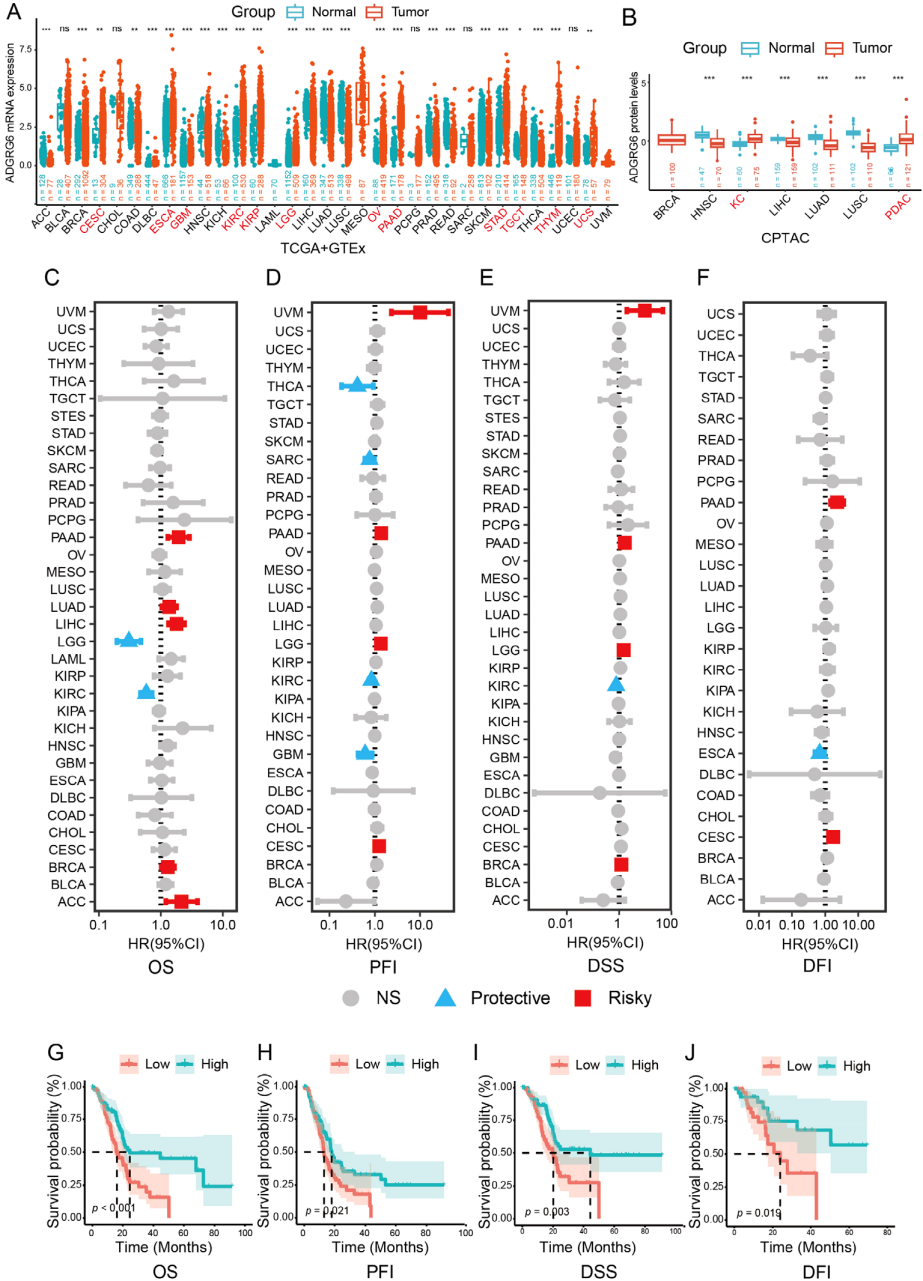
ADGRG6 served as a potential biomarker for PAAD. **(A)** Comparative analysis of ADGRG6 mRNA expression in normal versus tumor samples within the TCGA Pan-Cancer cohort. **(B)** Comparative assessment of ADGRG6 protein expression in normal and tumor samples from the CPTAC Pan-Cancer cohort. **(C-F)** Univariate Cox regression analysis elucidated the association between ADGRG6 expression and key survival metrics within the TCGA Pan-Cancer cohort: **(C)** overall survival (OS), **(D)** disease-specific survival (DSS), **(E)** progression-free interval (PFI), and **(F)** disease-free interval (DFI). **(G-J)** In the PAAD cohort, Kaplan-Meier plotters were utilized to assess the prognostic values of ADGRG6 across the same survival metrics: **(G)** OS, **(H)** PFI, **(I)** DSS, and **(J)** DFI. * p < 0.05; ** p < 0.01; *** p < 0.001.

To investigate the expression and potential tumor-regulatory role of ADGRG6, we analyzed expression data from the TCGA Pan-Cancer cohort and the GTEx database. The results revealed that ADGRG6 mRNA levels were elevated in several cancers when compared to the corresponding normal tissues, including CESC (cervical squamous cell carcinoma and endocervical adenocarcinoma), ESCA (Esophageal Cancer), GBM (Glioblastoma), KIRC (kidney renal clear cell carcinoma), KIRP (kidney renal papillary cell carcinoma), LGG (brain Lower Grade Glioma), OV (ovarian serous cystadenocarcinoma), PAAD (pancreatic adenocarcinoma), STAD (stomach adenocarcinoma), TGCT (testicular germ cell tumors), THYM (thymoma), and UCS (uterine carcinosarcoma) (**Figure 1A**). Additionally, CPTAC data showed that ADGRG6 protein expression was higher in tumors compared to normal tissues in KC (kidney cancer) and PDAC (pancreatic ductal adenocarcinomas) (**Figure 1B**).

Survival time is a crucial indicator for assessing tumor prognosis. To evaluate the prognostic value of ADGRG6, we performed univariate Cox analysis to examine its relationship with survival time across four metrics: overall survival (OS), progression-free interval (PFI), disease-specific survival (DSS), and disease-free interval (DFI). ADGRG6 was consistently associated with poor prognosis in PAAD, rather than other cancers, across all four evaluation methods (**Figures 1C–F**, **Supplementary Table S1–S4**). Kaplan-Meier curves further confirmed that ADGRG6 expression correlated with worse prognosis in the TCGA PAAD cohort (**Figures 1G–J**). Therefore, these results suggested that ADGRG6 could potentially serve as a relatively specific biomarker for PAAD.

### 3.2 Relationship between ADGRG6 and clinical characteristics in multiple PAAD cohorts

**Figure 2:**
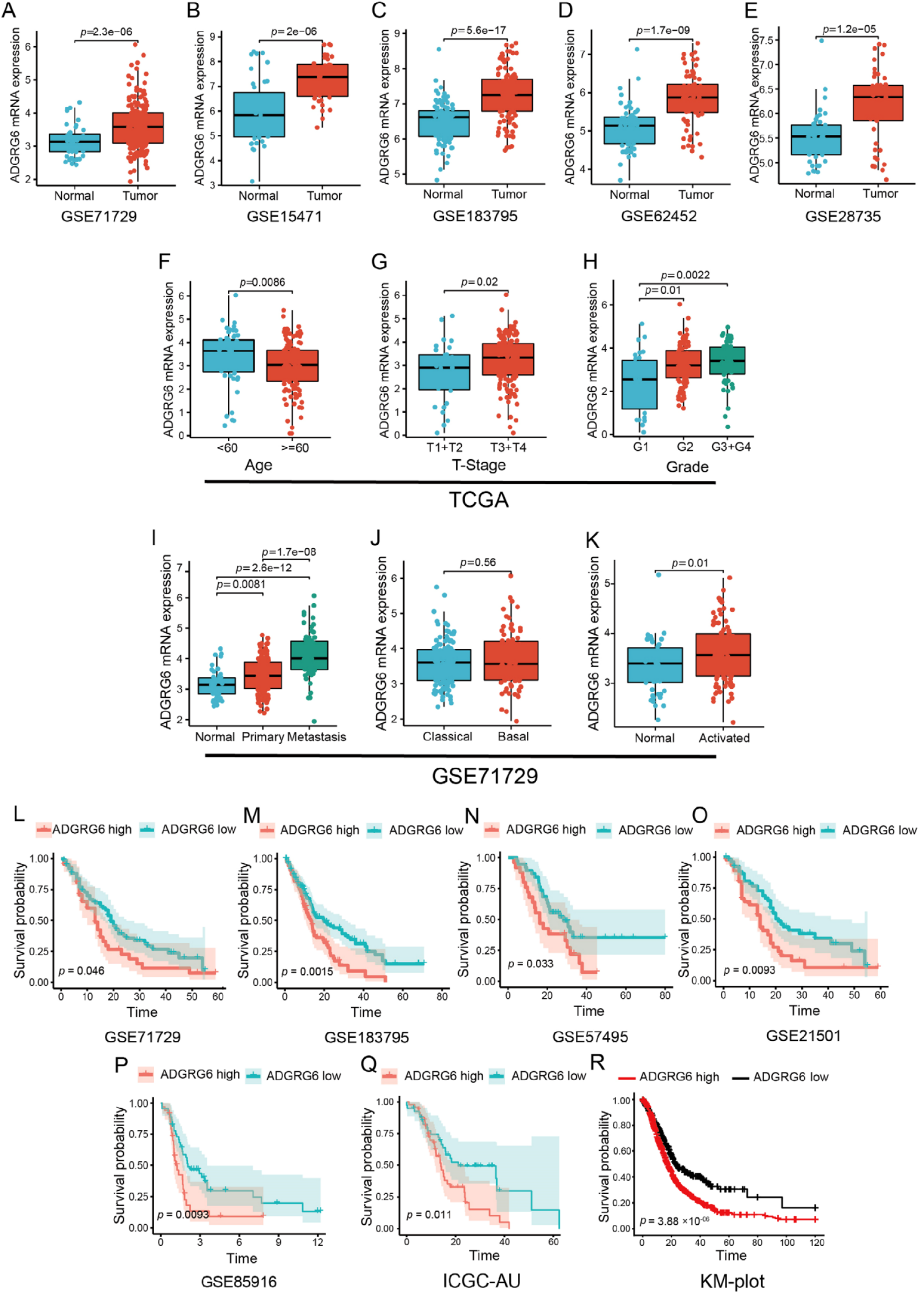
Exploration of ADGRG6 mRNA expression across multiple datasets revealed its correlations with clinical features and prognostic significance in PAAD. **(A-E)** Comparative analysis of ADGRG6 mRNA expression levels between normal and tumor samples across multiple cohorts from the GEO database: **(A)** GSE71729, **(B)** GSE15471, **(C)** GSE183795, **(D)** GSE62452, and **(E)** GSE28735. **(F-H)** Examination of ADGRG6 mRNA expression levels in relation to various clinical characteristics within the TCGA-PAAD cohort: **(F)** age, **(G)** tumor stage (T-stage), and **(H)** grade. **(I-K)** In the GSE71729 cohort, an analysis of ADGRG6 mRNA expression levels in different contexts: **(I)** metastasis status, **(J)** tumor-specific subtypes, and **(K)** stroma-specific subtypes. **(L-R)** Kaplan-Meier plotter assessments of the prognostic value of ADGRG6 expression across Overall Survival (OS) in various cohorts: **(L)** GSE71729, **(M)** GSE183795, **(N)** GSE57495, **(O)** GSE21501, **(P)** GSE85916, **(Q)** ICGC-AU, and **(R)** KM-plot cohort.

To further substantiate our findings regarding the potential oncogenic role of ADGRG6 based on the TCGA PAAD cohort, several independent PAAD cohorts from the GEO database were then utilized to validate the observation. Analysis of the GSE71729, GSE15471, GSE183795, GSE62452, and GSE28735 datasets revealed that ADGRG6 expression was significantly higher in tumor samples compared to normal pancreatic tissues (**Figures 2A–E**).

Further examination of the relationship between ADGRG6 mRNA expression and clinical characteristics showed that, in the TCGA PAAD cohort, ADGRG6 was more highly expressed in younger patients and those with higher T-stage and grade (**Figures 2F–H**), although no significant association was observed with N-stage or chronic pancreatitis status (**Supplementary Figure S1A, B**). In the GSE71729 cohort from the GEO database, ADGRG6 expression was notably higher in metastatic PAAD compared to primary tumors (**Figure 2I**). Tumor molecular typing, crucial for guiding molecular-targeted therapies, classified PAAD into “classical” and “basal-like” subtypes, with the latter generally associated with poorer prognosis (13). Despite this, no significant difference in ADGRG6 expression was found between these two subtypes (**Figure 2J**). Additionally, analysis based on stromal molecular signatures revealed that ADGRG6 expression was higher in the “activated” subtype, which is associated with macrophages, inflammation, and an activated fibroblast state, compared to the “normal” subtype (**Figure 2K**). Kaplan-Meier survival analysis across all validation cohorts (GSE71729, GSE183795, GSE57495, GSE21501, GSE85916, ICGC-AU, and KM-plot) demonstrated that high ADGRG6 expression was correlated with shorter survival (**Figures 2L–R**). These results collectively suggested that ADGRG6 held significant potential as a prognostic marker for PAAD.

### 3.3 Mutation and gene functional enrichment analysis of ADGRG6 in PAAD

**Figure 3:**
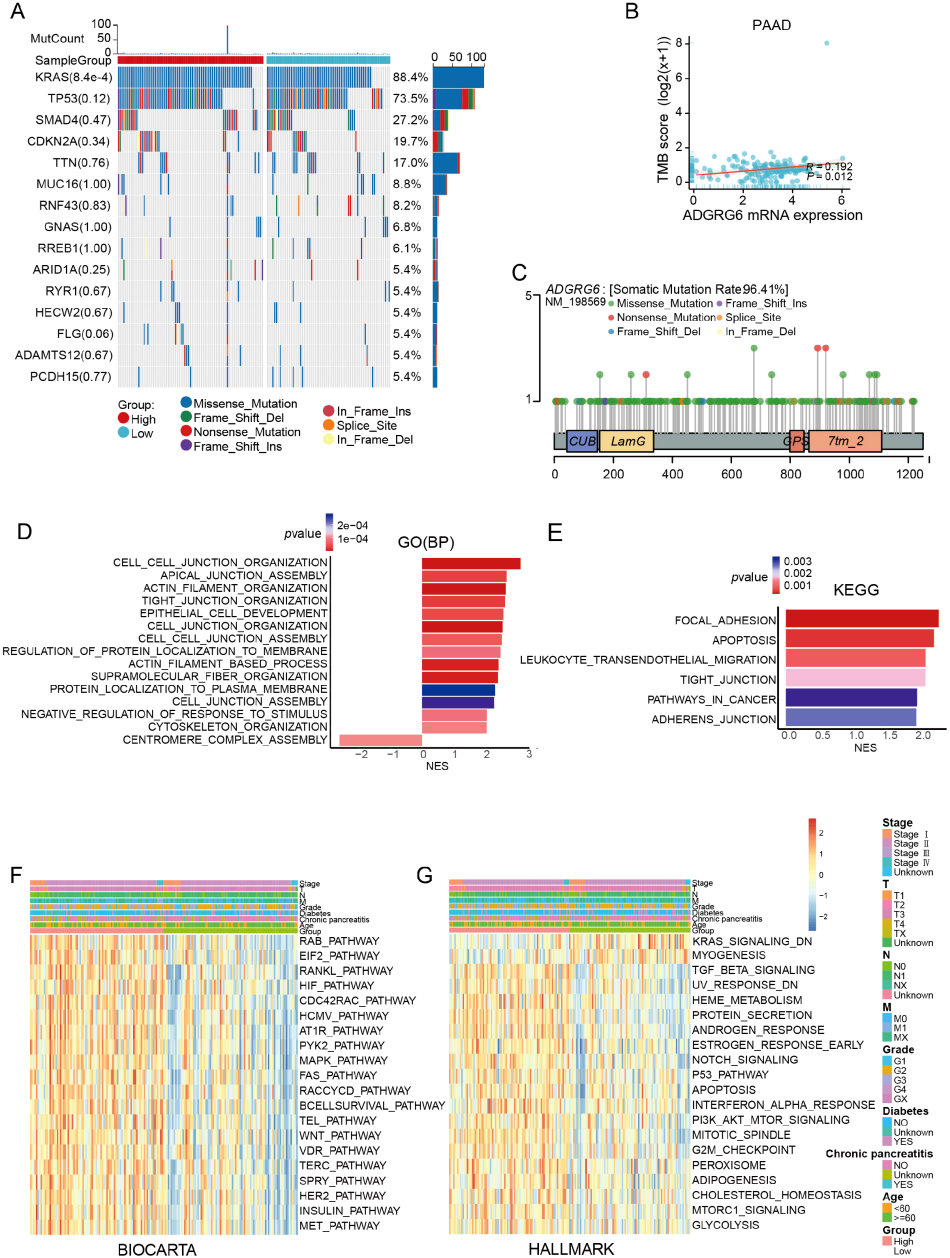
Mutation and gene functional enrichment analysis of ADGRG6 in PAAD. **(A)** Comparison of mutational landscapes between groups with high and low ADGRG6 expression in PAAD. **(B)** Scatter plot analysis depicting the correlation between ADGRG6 expression and TMB in pancreatic adenocarcinoma (PAAD). **(C)** A lollipop diagram illustrating the structural variants of the ADGRG6 protein within the TCGA Pan-Cancer cohort. **(D, E)** Gene Ontology (GO) and Kyoto Encyclopedia of Genes and Genomes (KEGG) pathway enrichment analyses delineated the functional roles of differentially expressed genes between the ADGRG6-high and ADGRG6-low groups. **(F, G)** Heatmaps displayed the Gene Set Variation Analysis (GSVA) scores for **(F)** BIOCARTA and **(G)** HALLMARK pathways, comparing the ADGRG6-high group with the ADGRG6-low group. Only pathways with statistically significant differences (*p* < 0.05) were presented.

Gene mutation is one of the main characteristics of tumors. We analyzed the relationship between ADGRG6 and common mutations in PAAD. The ADGRG6-high group notably exhibited a higher proportion of KRAS mutations (a common mutation in PAAD) (**Figure 3A**), suggesting a potential association between ADGRG6 and increased malignancy. Furthermore, ADGRG6 expression was positively correlated with tumor mutational burden (TMB) and microsatellite instability (MSI) across PAAD (**Figure 3B**, **Supplementary Figure S2A, B**). Lollipop charts illustrating ADGRG6 protein mutations in the TCGA pan-cancer cohort indicated that missense mutations were the predominant mutation type (**Figure 3C**). To further elucidate the potential role of ADGRG6 in PAAD, we performed gene set enrichment analysis (GSEA) by dividing the TCGA PAAD cohort into ADGRG6-high and -low groups based on median expression levels. ADGRG6 was associated with several extracellular matrix and migration-related pathways, including “cell-cell junction organization,” “actin filament organization,” “focal adhesion,” and “adherens junction” (**Figures 3D, E**). Gene set variation analysis (GSVA) scores indicated that the ADGRG6-high group had elevated scores for the p53 signaling pathway, WNT signaling pathway, MAPK signaling pathway, TGF-β signaling pathway, PI3K-AKT signaling pathway, and G2/M checkpoint pathway compared to the ADGRG6-low group (**Figures 3F, G**), suggesting a strong association with cancer growth and metastasis.

### 3.4 Immune infiltration analysis showed that ADGRG6 might be involved in immune escape of PAAD

**Figure 4:**
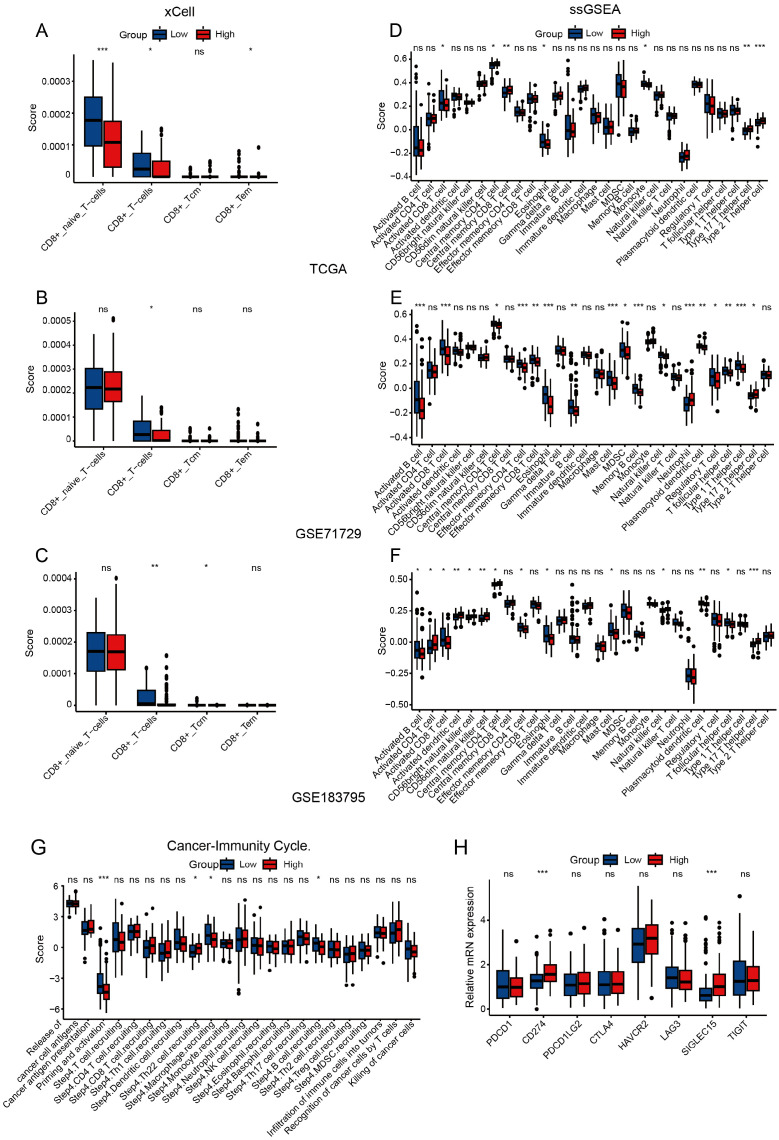
Assessment of immune infiltration. **(A-C)** Analysis of CD8^+^ T cells immune infiltration differences between the ADGRG6-high and ADGRG6-low groups using xCell in **(A)** TCGA, **(B)** GSE71729, and **(C)** GSE183795. **(D-F)** Analysis of immune infiltration differences between the ADGRG6-high and ADGRG6-low groups using ssGSEA in **(D)** TCGA, **(E)** GSE71729, and **(F)** GSE183795. **(G)** In TCGA, TIP (Tracking Tumor Immunophenotype) predicted the difference in tumor immune cycle status between ADGRG6-high group and ADGRG6-low groups. **(H)** Comparative analysis of immune checkpoint marker expression levels between the ADGRG6-high and ADGRG6-low groups. * p < 0.05; ** p < 0.01; *** p < 0.001.

Given the immunosuppressive nature of PAAD’s tumor microenvironment (27–29), we performed immune infiltration analysis using three independent PAAD datasets (TCGA, GSE71729, and GSE183795). CIBERSORT analysis showed that high ADGRG6 expression was associated with lower levels of naive B cells in all three groups (**Supplementary Figure S3A–C**). Although the role of naive B cells in the pancreatic cancer microenvironment has not been fully elucidated, some reports have indicated that high infiltration of B cells is associated with anti-tumor immunity and better prognosis (30–32). CD8^+^ T cells are essential for tumor immunity, as they can recognize and kill tumor cells (33). CIBERSORT analysis revealed a trend towards lower CD8+ T cell infiltration in the ADGRG6-high group across the three datasets, although this did not reach statistical significance (**Supplementary Figure S3A–C**). The xCell algorithm further demonstrated a significant decrease in CD8+ T cell infiltration in the ADGRG6-high group across all the three datasets (**Figures 4A–C**). Moreover, ssGSEA analysis showed that the ADGRG6-high expression group had a lower activated CD8^+^ T cell score but a higher Th17 score (**Figures 4D–F**). High Th17 infiltration may be related to tumor growth, pancreatic intraepithelial neoplasia (PanIN) formation, stem cell properties, and migration (34–36). Further exploration of the relationship between ADGRG6 expression and various stages of the tumor immune cycle revealed distinct immune activity levels between the two groups, particularly during the priming and activation stages. Notably, the high ADGRG6 expression group exhibited reduced T cell and APC activation (**Figure 4G**). Cancer cells can exploit immune checkpoints to evade immune responses, and the ADGRG6-high group displayed increased levels of CD274/PDL1 and SIGLEC15 expression (**Figure 4H**). These findings suggested that ADGRG6 might facilitate PAAD development by regulating immune infiltration, ultimately leading to immune escape.

### 3.5 Analysis of ADGRG6 expression pattern at the single-cell level in PAAD

**Figure 5:**
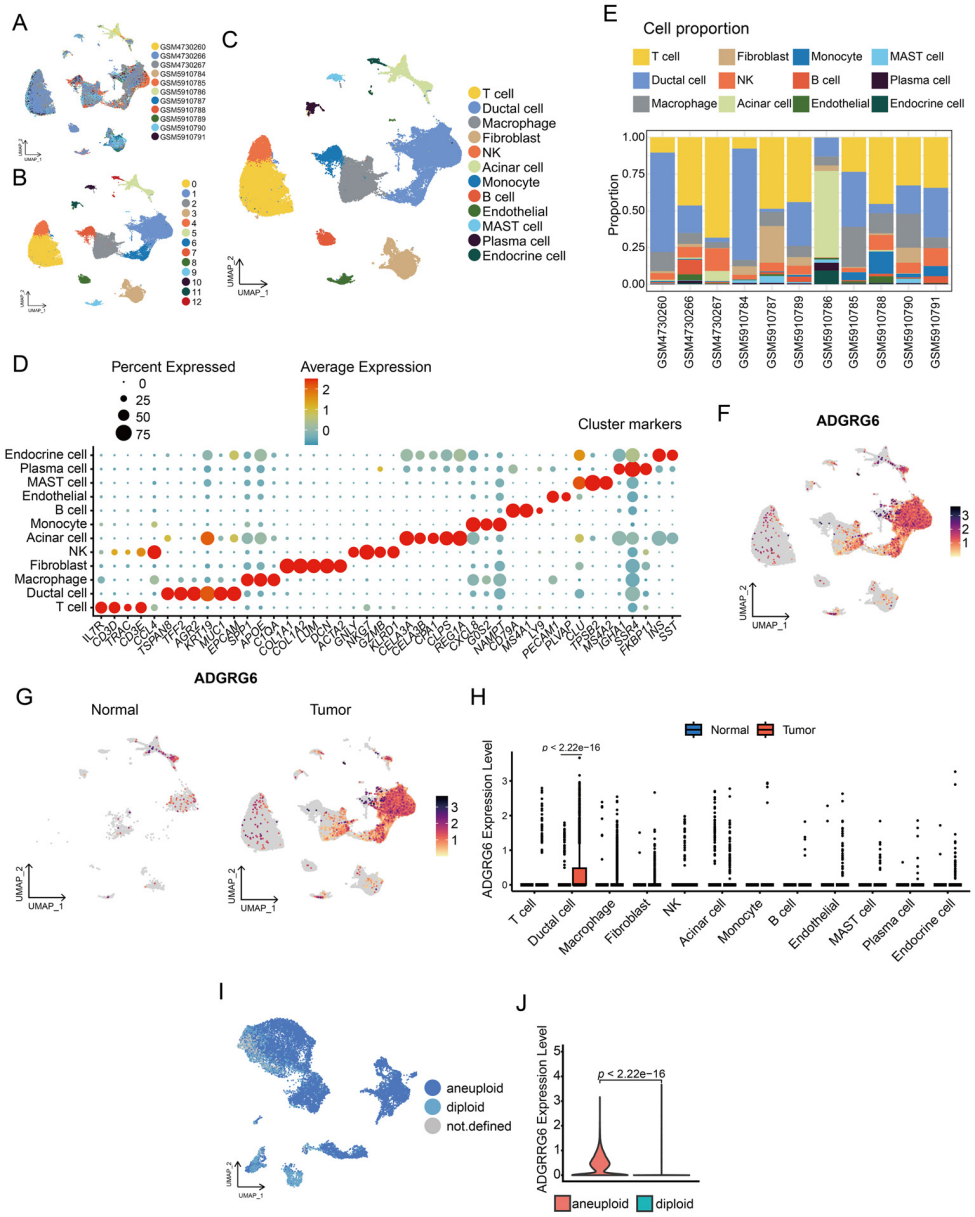
Single-cell analysis of ADGRG6 expression in PAAD. **(A-C)** A UMAP plot provided a visual representation of the single-cell landscape in PAAD, highlighting **(A)** 11 patient samples, **(B)** 13 cell clusters, and **(C)** 12 annotated cell types. **(D)** A dot plot showcased marker genes used for cell type annotation, with dot size indicating the proportion of cells expressing the gene and dot depth representing the relative expression level. **(E)** A bar graph illustrating the proportion of each cell type across different patient samples. **(F)** Feature plots displayed ADGRG6 expression across various cell types, allowing for a direct comparison of expression levels within the single-cell context. **(G, H)** Comparative analysis of ADGRG6 expression between PAAD and normal pancreas was presented in **(G)** a feature plot and **(H)** a violin plot. **(I)** CopyKat predictions for aneuploidy and diploidy in ductal cells of PAAD samples. **(J)** A comparative analysis of the relative expression level of ADGRG6 in aneuploid and diploid cells.

We obtained single-cell sequencing data from PAAD and corresponding normal pancreatic tissue samples from 11 patients, sourced from the GEO database (**Figure 5A**). After rigorous quality control, dimensionality reduction, and clustering, we identified 13 distinct cell subsets (**Figure 5B**). Further cell annotation led to the classification of 12 cell types, including T cells, ductal cells, macrophages, fibroblasts, natural killer (NK) cells, acinar cells, monocytes, B cells, endothelial cells, mast cells, plasma cells, and endocrine cells, which were visualized using UMAP (**Figure 5C**). The marker genes of each cell type following single-cell clustering, displayed in **Figure 5D**, served as the foundation for annotating the cell types. Additionally, we examined the cellular composition across samples. As indicated in **Figure 5E** and **Supplementary Figure S4**, GSM5910786, derived from normal pancreatic tissue, was predominantly composed of acinar and ductal cells; in contrast, PAAD tissues were rich in diverse immune cells and fibroblasts, highlighting the complexity of their tumor microenvironment.

For the gene ADGRG6, its expression was most pronounced in ductal cells (**Figure 5F**). A comparative analysis of normal and tumor tissues revealed significantly elevated ADGRG6 expression in the ductal cells of tumor samples compared to those in normal tissue (**Figures 5G, H**). Employing the COPYKAT algorithm, we detected aneuploidy in ductal cells within tumor samples, which we classified as malignant cells (**Figure 5I**). Intriguingly, ADGRG6 expression was significantly higher in these malignant cells compared to non-malignant cells within tumor samples (**Figure 5J**).

### 3.6 Prediction of ligand-receptor interactome reveals the microenvironmental regulatory mechanism potentially involving ADGRG6

**Figure 6:**
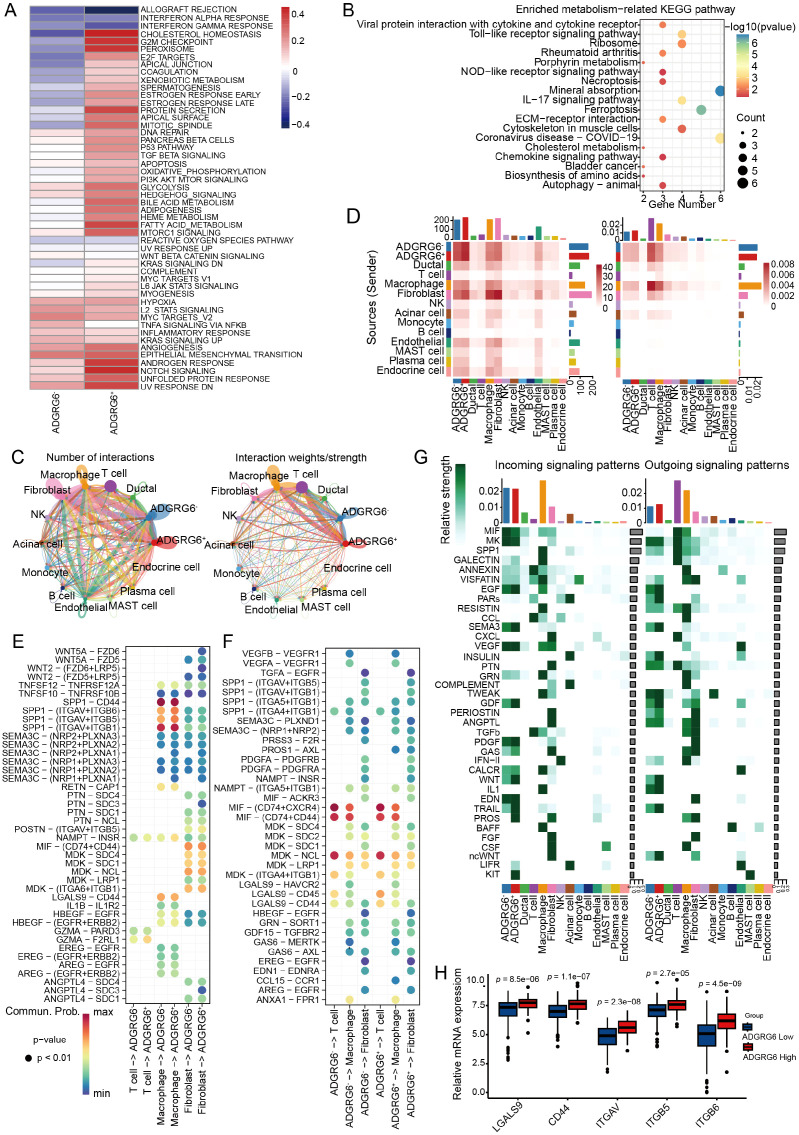
Exploring the regulatory impact of ADGRG6 in the PAAD microenvironment. **(A)** A heatmap comparison of Gene Set Variation Analysis (GSVA) scores for hallmark pathways between ADGRG6^+^ and ADGRG6^-^ malignant cells. **(B)** KEGG enrichment analysis results of differentially expressed genes between ADGRG6^+^ malignant cells and ADGRG6^-^ malignant cells. **(C)** A network diagram illustrating the complex intercellular interactions within the PAAD microenvironment. **(D)** A heatmap displaying the intersection number and weights/strength of interactions between ADGRG6^+/-^ malignant cells and other cell types, offering quantitative insights into the cellular crosstalk. **(E, F)** Bubble plots representing the strength of ligand-receptor interactions: **(E)** Input and **(F)** Output. The size of the points corresponded to the significance of the interaction (smaller points indicate larger *p*-values), while the darkness of the color signified the strength of the interaction. **(G)** An overview of the main input and output signaling pathways for each cell subset, highlighting the key pathways driving cellular communication in the PAAD microenvironment. **(H)** Comparative analysis of ADGRG6-related ligand-receptor expression levels between the ADGRG6-high and ADGRG6-low groups within the TCGA-PAAD cohort.

We designated malignant cells expressing ADGRG6 as ADGRG6^+^ malignant cells and those without expression as ADGRG6^-^ malignant cells. The ADGRG6^+^ group exhibited higher scores in the P53 pathway, G2/M checkpoint, TGF-β signaling, PI3K-AKT signaling, and oxidative phosphorylation pathways (**Figure 6A**), corroborating our previous analysis of the TCGA PAAD dataset (**Figures 4C, D**). Moreover, differential gene enrichment analysis between the two groups indicated that ADGRG6 was associated with pathways such as Toll-like receptor signaling, mineral absorption, necroptosis, ferroptosis, and ECM-receptor interaction (**Figure 6B**).

Utilizing the “CellChat” platform, we visualized ligand-receptor (L-R) mediated cell-cell interactions, revealing a higher frequency of interactions between the ADGRG6^+^ group and other cells, notably fibroblasts and macrophages (**Figures 6C, D**). We conducted a detailed analysis of the L-R interaction strength between ADGRG6^-^ and ADGRG6^+^ malignant cells and their counterparts among T cells, macrophages, and fibroblasts, both in terms of input and output signals (**Figures 6E, F**). These interactions were depicted in bubble plots for clarity. Secreted phosphoprotein 1 (SPP1), an integrin-binding protein, is released by various cell types, including macrophages, endothelial cells, and osteoclasts (37). We observed that the interaction strength of the SPP1-(ITGAV+ITGB5/ITGB6) axis between macrophages and ADGRG6^+^ malignant cells surpassed that of the ADGRG6^-^ group; likewise, the frequency of LGALS9-CD44/CD45 interactions was higher in the ADGRG6^+^ group (**Figures 6E, F**). LGALS9 is known to enhance the function and stability of induced T regulatory cells (iTregs), promote the expression of Foxp3 in iTregs, and suppress the activation of CD8^+^ T cells, thereby contributing to an immunosuppressive state (38). Further analysis of the input and output signaling patterns among different cell types revealed significant differences in the transmission of SEMA3, GDF, WNT, GAS, TRAIL, PARs, IL1, and other signals between the two groups (**Figure 6G**). To highlight the differential L-R interactions, we presented the expression levels of these genes in each cell type. Consistent with our findings, the genes showed higher expression levels in the ADGRG6^+^ group as compared to the ADGRG6^-^ group (**Figure 6H**).

### 3.7 ADGRG6 promotes the growth and metastasis of PAAD

**Figure 7:**
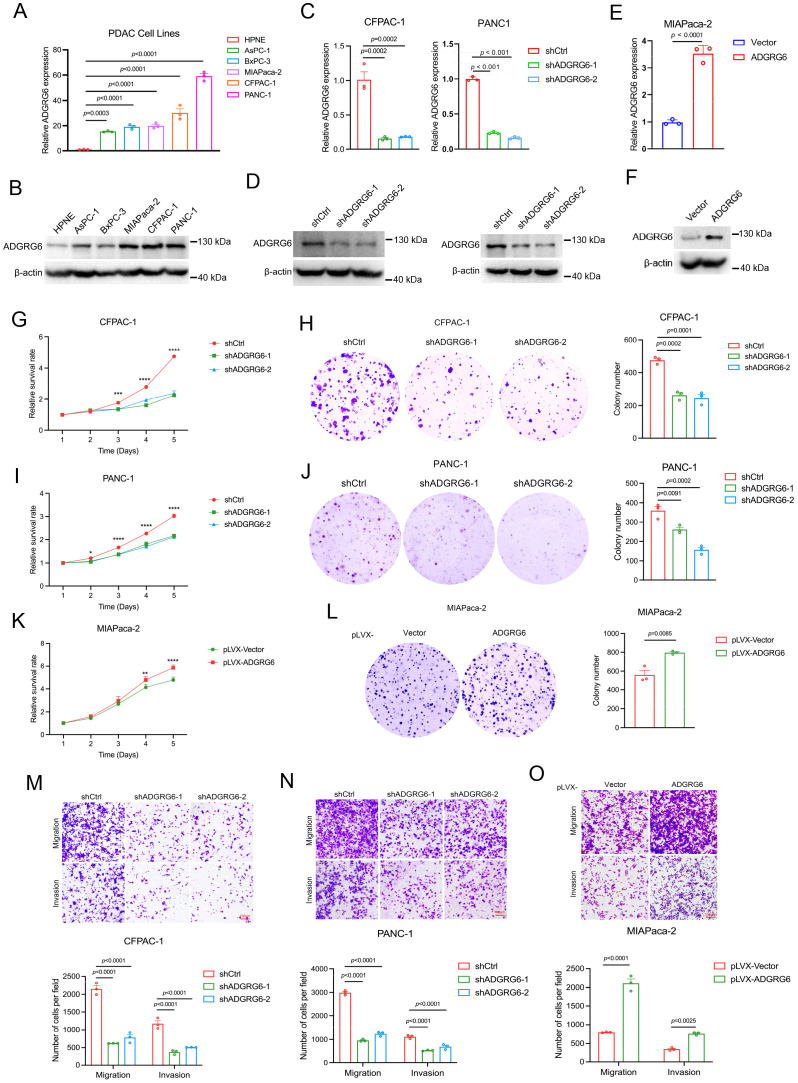
Effects of ADGRG6 on proliferation and metastasis of PAAD cells. **(A, B)** Expression of ADGRG6 mRNA **(A)** and protein level **(B)** in human pancreatic epithelial cell line hTERT-HPNE and various PAAD cell lines. **(C-F)** The efficiencies of ADGRG6 knockdown in CFPAC-1 and PANC-1, and overexpression in MIAPaca-2 were detected by real-time PCR **(C, E)** and Western blot **(D, F)**. **(G-K)** The influence of ADGRG6 knockdown on proliferation of CFPAC-1 and PANC-1 cells and ADGRG6 overexpression on that of MIAPaca-2 were evaluated by MTT **(G, I, K)** and colony formation **(H, J, L)** assays. **(M, N)** The transwell assay was used to explore the influence of ADGRG6 on the metastasis of PDAC cells. Data represented the mean + SEM (n = 3, three independent experiments), and the *p* values were analyzed by one-way ANOVA **(A, C, G, H, I, J, M, N)** or unpaired two-tailed Student’s t test **(E, K, L, O)**. * p < 0.05; ** p < 0.01; *** p < 0.001, **** p < 0.0001.

To investigate the role of ADGRG6 in PAAD, we first analyzed its mRNA and protein levels in several PAAD cell lines. Compared to the human pancreatic duct epithelial cell line hTERT-HPNE, both the mRNA and protein expression levels of ADGRG6 were significantly upregulated in all the five PAAD cell lines, including AsPC-1, BxPC-3, MIAPaca-2, CFPAC-1, and PANC-1 (**Figures 7A, B**). We then knocked down ADGRG6 in ADGRG6-high CFPAC-1 and PANC-1 cells, while overexpressed it in ADGRG6-low MIAPaca-2 cells (**Figures 7C, F**). MTT and colony formation assays demonstrated that ADGRG6 knockdown reduced the proliferation of CFPAC-1 and PANC-1 cells (**Figures 7G–J**). Conversely, ADGRG6 overexpression enhanced the growth of MIAPaca-2 cells (**Figures 7K–L**). We also investigated the impact of ADGRG6 on the metastatic potential of PAAD cells. Transwell assays were conducted to assess the migration and invasion capabilities of PAAD cells following ADGRG6 knockdown and overexpression. The results showed that ADGRG6 knockdown significantly reduced the migration and invasion abilities of CFPAC-1 and PANC-1 cells (**Figures 7M, N**), while ADGRG6 overexpression enhanced those of MIAPaca-2 cells (**Figure 7O**). These results indicated that ADGRG6 promotes the growth, migration and invasion of PAAD cells.

**Figure 8:**
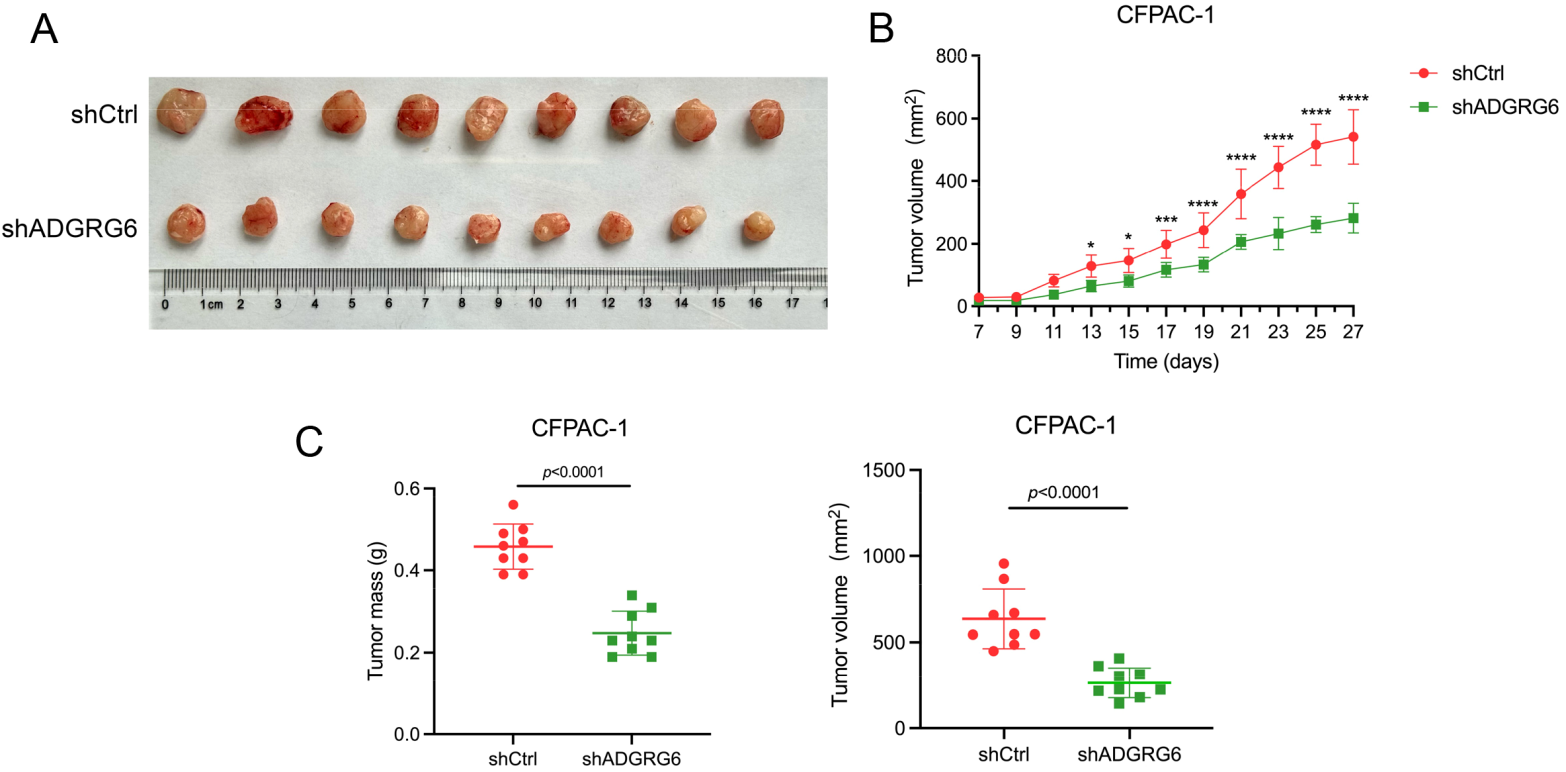
ADGRG6 promotes the growth of subcutaneous xenografts of PAAD. We administered injections to BALB/c nude mice with either shCtrl or shADGRG6 CFPAC-1 cells, at a dosage of 1 million cells per mouse. Monitoring of tumor growth commenced on day 7 following the injections, and the tumors were collected on day 27. **(A)** Image of harvested transplanted tumors on day 27. **(B)** Tumor growth curve. **(C)** Comparison of volume/weight of control/knockdown groups on day 27 (n = 9 per group). * p < 0.05; ** p < 0.01; *** p < 0.001, **** p < 0.0001.

To assess the impact of ADGRG6 knockdown on the growth of subcutaneously transplanted pancreatic tumors in a mouse model, we injected BALB/c nude mice with either control or ADGRG6 knockdown CFPAC-1 cells (1 × 10^6^ cells per mouse). We began monitoring tumor size from day 7 post-injection, and the tumors were harvested at the end of observation. Our results demonstrated a noticeable reduction in both the volume and weight of the subcutaneous tumors in the mice that received ADGRG6 knockdown cells as compared to the control group (**Figures 8A-C**).

Together, these findings provide evidence supporting the role of ADGRG6 in promoting PAAD progression.

### 3.8 ADGRG6 regulates mutated p53 and its related pathways in PAAD

**Figure 9:**
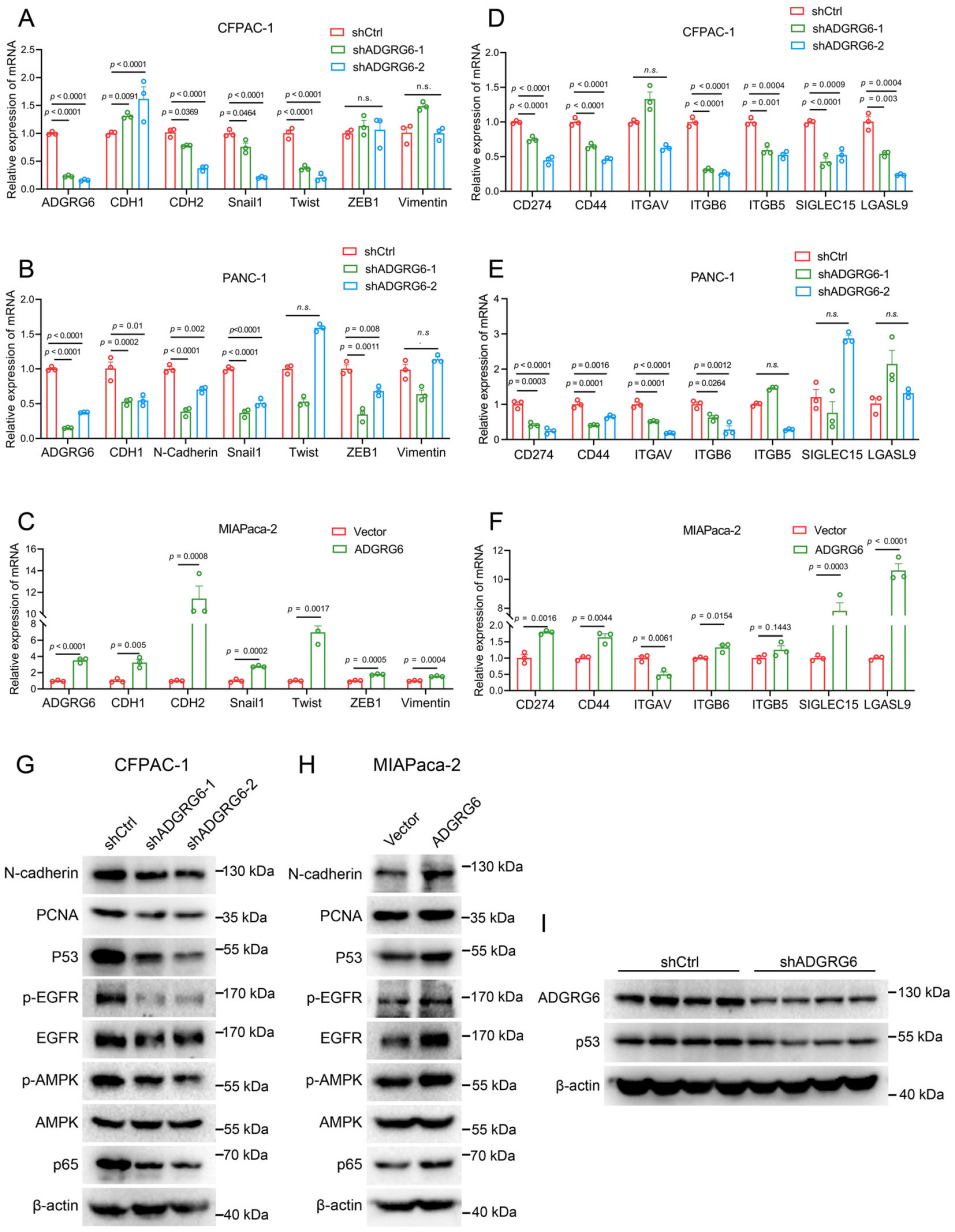
ADGRG6 regulates mutated p53 and its related pathways in PAAD. **(A-C)** Effect of ADGRG6 knockdown or overexpression on EMT-related marker genes were detected by real-time PCR in **(A)** CFPAC-1, **(B)** PANC1 and **(C)** MIApaca-2 cell lines. **(D-F)** Real-time PCR detection of the effect of ADGRG6 knockdown or overexpression on immune- and matrix-related genes in **(D)** CFPAC-1, **(E)** PANC1 and **(F)** MIAPaca-2 cell lines. **(G, H)** Effect of ADGRG6 knockdown or overexpression on the signaling pathways involved in the GOF of mutp53 were detected by Western blot in **(G)** CFPAC-1 and **(H)** MIAPaca-2. **(I)** Western blot showed the protein expression levels of ADGRG6 and p53 in shCtrl or shADGRG6 tumor tissues. Data represented the mean + SEM (n = 3, three independent experiments), and the *p* values were analyzed by one-way ANOVA **(A, B, D, E)** or unpaired two-tailed Student’s t test **(C, F)**.

We then investigated how ADGRG6 regulated the progression of PAAD. Utilizing real-time PCR, we observed a reduction in the expression of EMT-related marker genes following ADGRG6 knockdown in CFPAC-1 and PANC-1 cells, with a converse effect observed upon its overexpression in MIAPaca-2 cells, most notably for CDH2 (N-Cadherin) as depicted in **Figures 9A–C**. Similarly, the knockdown of ADGRG6 in CFPAC-1 and PANC-1 cells led to a decrease in the expression of various immune-related genes, including PDL1 (CD274), CD44, ITGAV, ITGB5, ITGB6, SIGLEC15, and LGASL9, while overexpression of ADGRG6 increased the expression of these genes in MIAPaca-2 cells (**Figures 9D–F**).

It is well established that wild-type p53 functions as a tumor suppressor, whereas most of the mutated p53 exhibit gain-of-function (GOF) activities, such as promoting cell proliferation, metastasis and evading immune surveillance (39–41). Notably, the p53 gene mutation frequency in PAAD exceeds 70% (9, 10). Using the CCLE database, we identified specific mutations of p53 in the three cell lines—CFPAC-1: C242R, PANC-1: R273H, MIAPaCa-2: R248W—that were associated with gain-of-function activities (42), as presented in **Supplementary Figure S5A**. Previous research has shown that p53 knockdown can reduce the migration and invasion capabilities of CFPAC-1 and PANC-1 cells (43). Interestingly, our western blot analysis demonstrated that knockdown of ADGRG6 resulted in a decrease in p53 protein levels in CFPAC-1 cells, while ADGRG6 overexpression had the opposite effect in MIAPaCa-2 (**Figures 9G, H**). Similarly, *in vivo* experiments showed similar results, the knockdown effect of ADGRG6 and the consequent decrease in p53 protein levels in the transplanted tumors were further validated by western blot analysis (**Figure 9I**, **Supplementary Figure S5B**). Furthermore, in these cell lines, we uncovered that ADGRG6 activated EGFR, AMPK and NF-κB signaling pathways (**Figure 9G, H**), which are believed to be modulated by mutated p53 protein to exert its GOF and work as the upstream molecules for the regulation of the proliferation-, EMT- and immune-related genes mentioned above (39, 44–47).

### 3.9 ADGRG6 regulates PAAD development through gain-of-function of mutated p53

**Figure 10:**
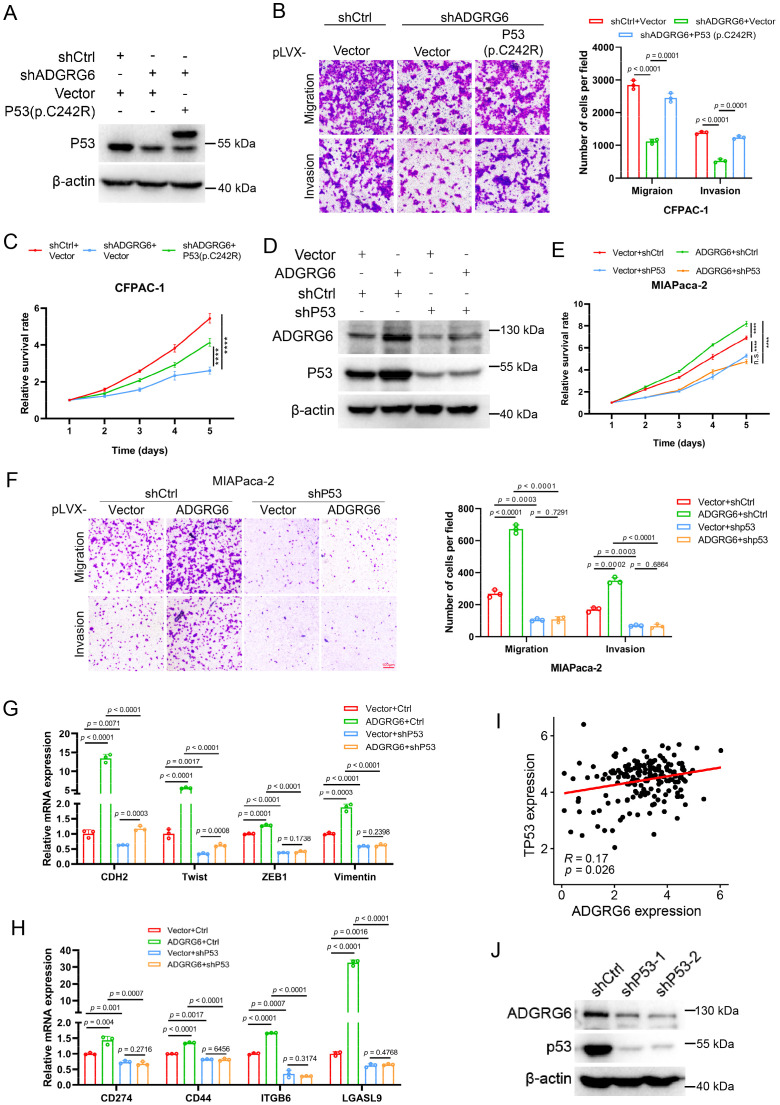
ADGRG6 regulates PAAD progression through mutp53. **(A)** Western blot showed the complementary effect of p53(C242R) in CFPAC-1 cells with ADGRG6 knockdown. **(B, C)** Effects of ADGRG6 knockdown on the growth, migration and invasion of CFPAC-1 cells were rescued by the forced expression of p53(C242R). **(D)** Western blot showed the knockdown effect of P53 in MIAPaca-2 cells overexpressing ADGRG6. **(E-H)** Knockdown of p53 weakened the effects of overexpression of ADGRG6 on the growth, migration, invasion, and expression of immune markers of MIAPaca-2 cells. **(I)** Correlation between the mRNA expression levels of ADGRG6 and p53. **(J)** Western blot showed that knockdown of P53 in MIAPaca-2 cells led to downregulation of ADGRG6 protein levels. Data represented the mean + SEM (n = 3, three independent experiments), and the *p* values were analyzed by one-way ANOVA **(B, C, E, F)**.

To further explore the potential role of the ADGRG6-mutated p53 signaling axis in pancreatic cancer, by constructing a CFPAC-1 cell model in which mutated p53 (C242R) was reintroduced following ADGRG6 knockdown, we observed that the inhibition of proliferation, migration, and invasion caused by ADGRG6 knockdown was substantially reversed by the overexpression of exogenous p53(C242R) (**Figures 10A–C**). Conversely, when we overexpressed ADGRG6 in MIAPaca-2 cells with endogenous p53 (mutp53) knockdown beforehand, the regulatory effects of ADGRG6 overexpression on proliferation, migration, invasion and the expression of the proliferation-, EMT- and immune-related markers of PAAD cells were greatly impaired when endogenous p53 was knocked down, suggesting that ADGRG6 mediated these effects through mutated p53 in PAAD cells (**Figures 10D–H**). These results demonstrated the pivotal role of the ADGRG6-mutated p53 signaling axis in facilitating the progression of PAAD.

Interestingly, we also found that ADGRG6 mRNA expression was elevated in samples harboring mutated p53 compared to those with wild-type p53 as well as its mRNA levels showed a positive correlation with p53 in the TCGA PAAD cohort (**Figure 10I**, **Supplementary Figure S6**). Furthermore, we found that ADGRG6 expression was significantly downregulated after knockdown of p53 in MIAPaca2 cells (**Figure 10J**), indicating a positively feedback loop of ADGRG6 and mutp53.

## 4 Discussion

PAAD develops rapidly and has a notoriously poor prognosis, characterized by high invasiveness, early metastasis, a complex tumor microenvironment, and resistance to chemotherapy and radiotherapy (1–5). Finding effective treatments for this aggressive cancer remains a significant challenge, underscoring the need for new therapeutic targets. While ADGRG6 has been extensively studied in neurodevelopment, its role in cancer is documented in colon, breast, and bladder cancers compared to its unexplored role in PAAD (24–26). In this report, we first summarized the expression pattern of ADGRG6 across pan-cancer. Utilizing data from the TCGA, GTEx, HPA, CPTAC, and CCLE databases, we discovered abnormal mRNA and protein expression of ADGRG6 in several cancers including PAAD. Survival and epigenetic analyses using the TCGA PAAD cohort strikingly suggested an oncogenic role of ADGRG6 in PAAD. Multi-cohort validation indicated a significant association between ADGRG6 and T stage, grade, metastasis, and stromal status, proposing its potential as a novel prognostic marker for PAAD. To explore the specific mechanisms of ADGRG6 in PAAD, we conducted gene enrichment and pathway scoring analyses, revealing strong correlations with pathways related to the extracellular matrix, cell adhesion, cytoskeleton, and cell cycle. These findings suggest a potential role for ADGRG6 in the proliferation and metastasis of PAAD.

ADGRG6 expression was higher in “activated” stromal samples, which were linked to worse prognosis and might indicate activated inflammatory responses. Notably, cell interaction analysis revealed a strong correlation between ADGRG6 and integrin ligand-receptor interactions related to cell-matrix adhesion, suggesting that ADGRG6 might play a role in regulating the stroma in PAAD. An important feature of PAAD is its immunosuppressive environment (13). The unique state of the extracellular matrix may contribute to this immunosuppression (27, 28, 48). We further explored the relationship between ADGRG6 and the immune microenvironment of PAAD. Our findings revealed that ADGRG6 was associated with lower infiltration of navie B cells and CD8+ T cells, along with a reduced immune activation score. Conversely, higher levels of Type 17 T helper cells (Th17) were observed in ADGRG6-high group. Activated CD8+ T cells are known for their role in directly killing tumors, while Th17 cells may indirectly influence tumor proliferation by modulating CD8+ T cell and macrophage function (49–52). Single-cell sequencing data corroborated these results, indicating that high expression of ADGRG6 correlated with the increased interactions of LGALS9 with CD44/CD45 between ductal cells and T cells, which promoted iTreg differentiation and inhibited CD8+ T cell activation (38). Furthermore, several reports have linked integrins to tumor metastasis and immune escape (53–57), and we identified a potential relationship between ADGRG6 and integrins. Subsequent RT-qPCR verification demonstrated that knocking down or overexpressing ADGRG6 altered the expression of these related genes. Therefore, we hypothesize that ADGRG6 was associated with immunosuppression in PAAD and could serve as a potential indicator for immunotherapy.

We then conducted cellular and animal experiments, which revealed that ADGRG6 promoted proliferation, migration, and invasion abilities of PAAD cell lines. Furthermore, we discovered that ADGRG6 significantly upregulated protein levels of p53, whose mutation frequency exceeds 70% in PAAD. Mutated p53 not only lost its anti-cancer effects but might also exhibits various GOF activities, which promote tumor proliferation, migration, metabolic reprogramming, and immune escape, influencing tumor epithelial-mesenchymal transition (EMT), immune checkpoints (such as PDL1), and stemness markers (such as CD44) through sigaling pathways such as EGFR, AMPK, and NF-κB (58–60). Consistently, we observed that ADGRG6 regulated the phosphorylation of EGFR and expression levels of p65 protein and EMT-related genes. We further found that the regulatory effects of ADGRG6 overexpression on proliferation, migration, invasion and the expression of the EMT- and immune-related markers of PAAD cells was mediated by mutated p53. Despite numerous studies aimed at targeting mutant p53 to inhibit tumors (61), no drug has successfully been marketed to date. Given that ADGRG6 is a G protein-coupled receptor (GPCR) situated on the cell membrane, identifying specific inhibitors targeting this receptor could emerge as a powerful strategy for curbing the progression of PAAD driven by mutated p53.

During the detection of the EMT markers regulated by ADGRG6 knockdown or overexpression, we observed that CDH1 and CDH2 were regulated in the opposite tendency in CFPAC-1 cells while in the same trend in PANC-1 and MIAPaca-2 cells (**Figures 9A–C**). Typically, CDH1 (E-cadherin) is dysregulated in cancers, with its downregulation often associated with increased tumor invasiveness and metastasis. Conversely, upregulation of CDH2 (N-cadherin) is commonly linked to the epithelial-mesenchymal transition (EMT), a key process that enables cancer cells to acquire an invasive phenotype. However, this change does not always correspond to metastatic ability. For instance, some studies have reported the coexistence of both markers in the transitional EMT state (62), suggesting that CDH1 is not always necessary for tumor EMT (63). Additionally, some cancer cells acquire mesenchymal traits while retaining epithelial markers (64). The mesenchymal transition of tumors is a multifaceted process, and it may not be fully explained by the inverse regulation of CDH1 and CDH2 alone. We believe that the ultimate impact on tumor invasiveness likely depends on the relative dominance of the epithelial or mesenchymal states in a given context.

In conclusion, our research has identified ADGRG6 as a novel prognostic marker and therapeutic target for PAAD. However, several limitations of this study warrant further investigation. Firstly, while our cell-based complementation model provides valuable preliminary insights into the role of the ADGRG6-mutant p53 signaling axis in regulating immune-related markers such as CD274, CD44, ITGB6, and LGALS9 in PAAD cells, it is essential to establish a mouse model with intact immune function to conduct relevant immunological experiments, which is crucial for confirming the role of the ADGRG6-mutant p53 axis in promoting immune escape in PAAD. Secondly, the molecular mechanisms underlying ADGRG6’s regulation of mutant p53 protein expression remain to be fully elucidated. Future studies should focus on addressing these gaps to enhance our understanding of the ADGRG6-mutant p53 axis and its potential as a therapeutic target in PAAD.

## Data Availability

Publicly available datasets were analyzed in this study. This data can be found here: TCGA,GTEX, https://xenabrowser.net/datapages/cProSite, https://cprosite.ccr.cancer.gov/cBioPortal, https://www.cbioportal.org/HPA, https://www.proteinatlas.org/CCLE, https://sites.broadinstitute.org/ccle/GEO, https://www.ncbi.nlm.nih.gov/geo/(GSE71729, GSE15471, GSE183795, GSE62452, GSE28735, GSE57495, GSE21501, and GSE85916) ICGC, https://dcc.icgc.org KM-plot, https://kmplot.com/analysis/index.php?p=service.
