# Supplementary material for: Identification of a novel prognostic marker ADGRG6 in pancreatic adenocarcinoma: multi-omics analysis and experimental validation
Source: Front Immunol. 2025 Mar 27;16:1530789. doi: 10.3389/fimmu.2025.1530789 (PMC11986822; doi:10.3389/fimmu.2025.1530789)
Supplement: Supplementary file 1 [file DataSheet1.docx]

**Supplementary Figures**


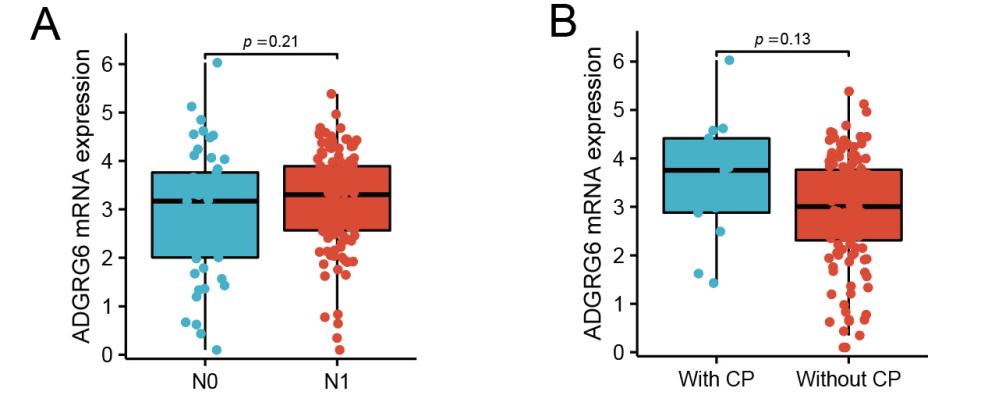


**Supplementary Figure S1 | Association of ADGRG6 expression with clinical features and mutational landscape.** Examination of ADGRG6 mRNA expression levels in relation to various clinical characteristics within the TCGA-PAAD cohort: **(A)** node stage (N-stage) and **(B)** chronic pancreatitis (CP) status.


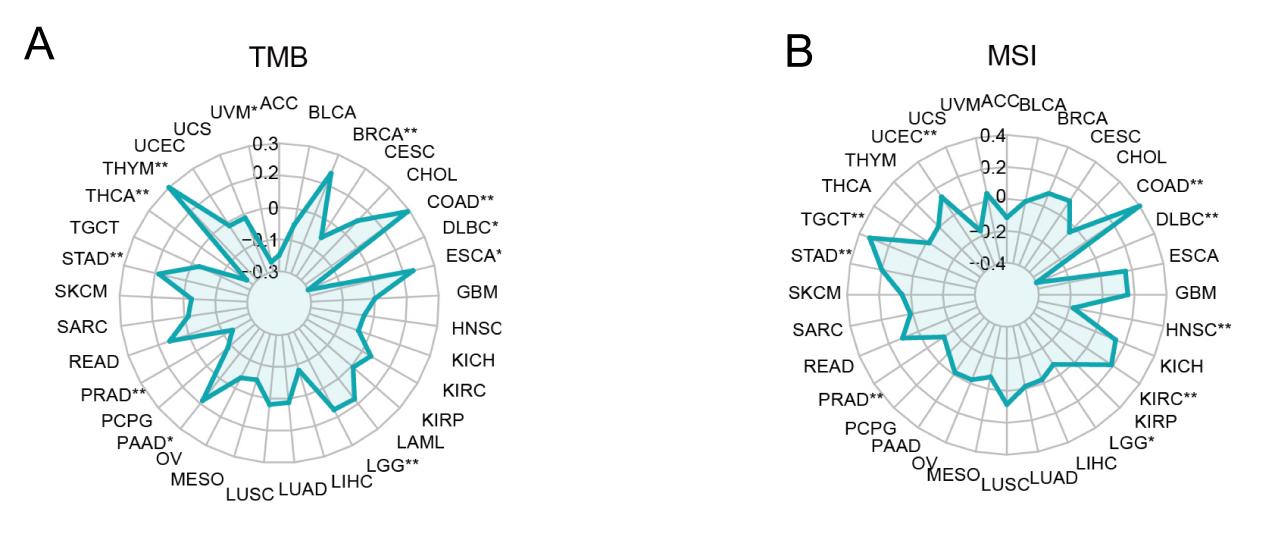


**Supplementary Figure S2 | Association of ADGRG6 expression with TMB and MSI in PAAD. (A)** Exploration of the correlation between ADGRG6 mRNA expression and tumor mutational burden (TMB). **(B)** Analysis of Microsatellite Instability (MSI) in relation to ADGRG6 expression.


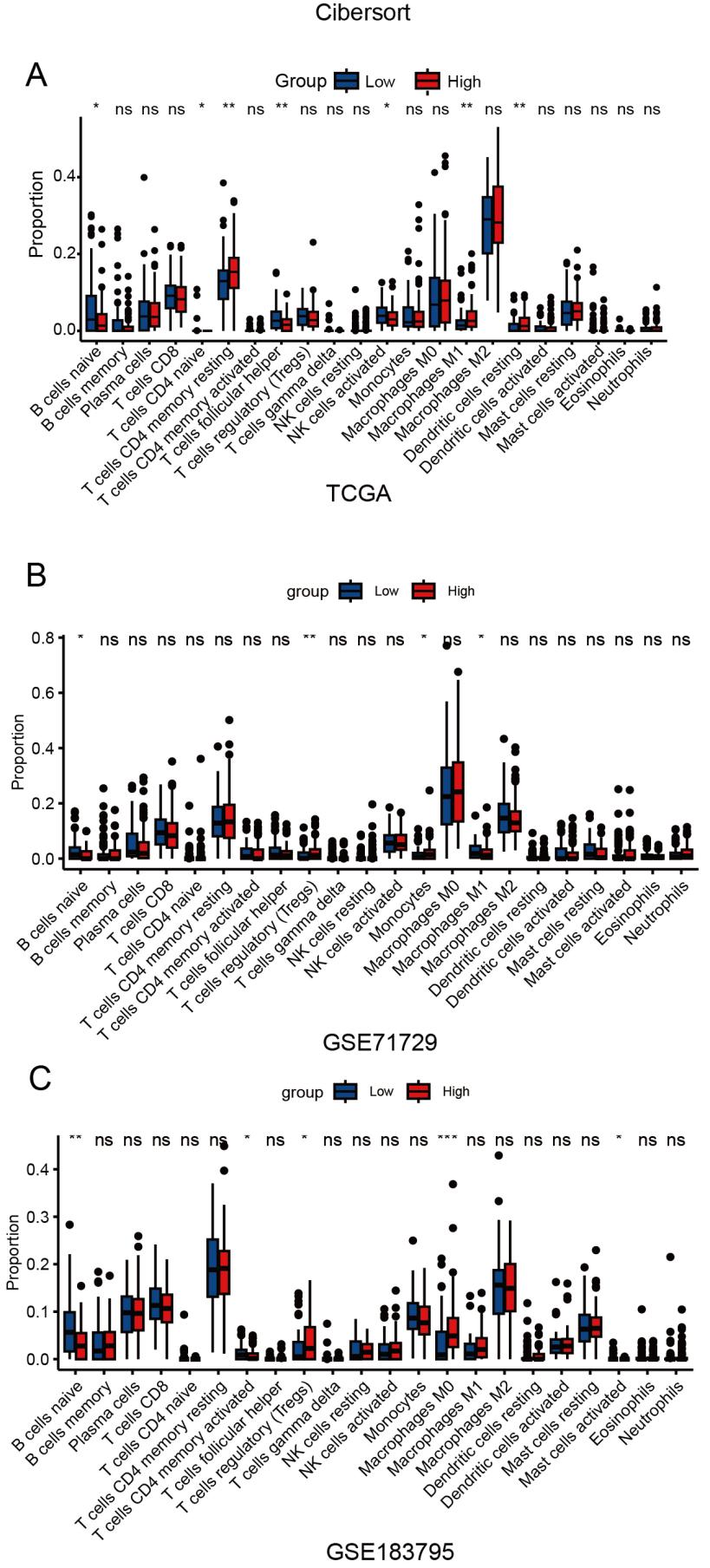


**Supplementary Figure S3 | The relationship between ADGRG6 expression and immune infiltration was determined using the Cibersort algorithm. (A-C)** Analysis of immune infiltration differences between the ADGRG6-high and ADGRG6-low groups using Cibersort in **(A)** TCGA, **(B)** GSE71729, and **(C)** GSE183795.


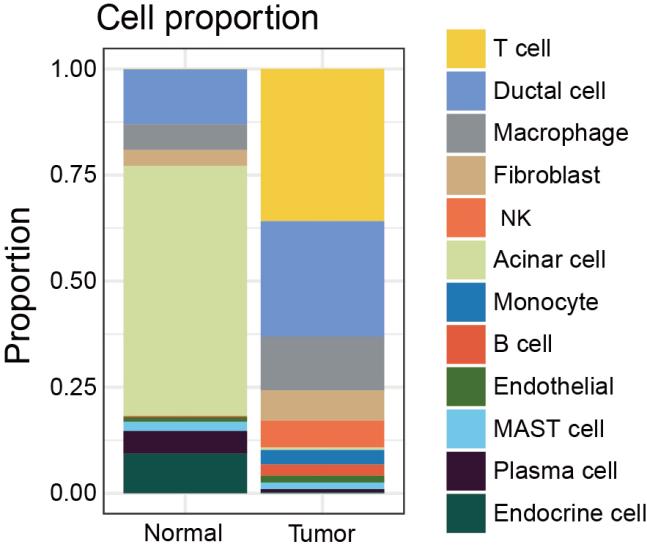


**Supplementary Figure S4 | The bar graph showed the proportion of each cell type in normal pancreatic tissue versus pancreatic cancer tissue.**


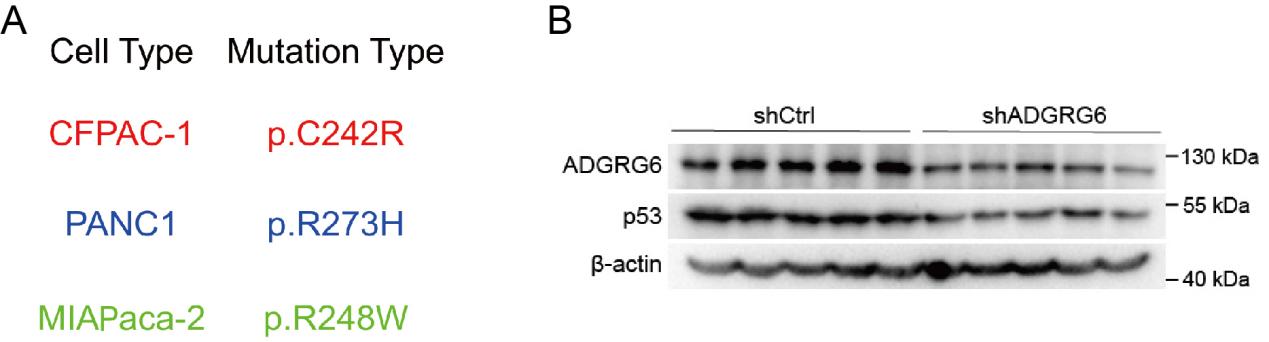


**Supplementary Figure S5 | (A)** Specific mutations of p53 in CFPAC-1, PANC-1 and MIAPaCa-2 were analyzed using the CCLE database. **(B)** Western blot showed the protein expression levels of ADGRG6 and p53 in shCtrl or shADGRG6 tumor tissues.

**
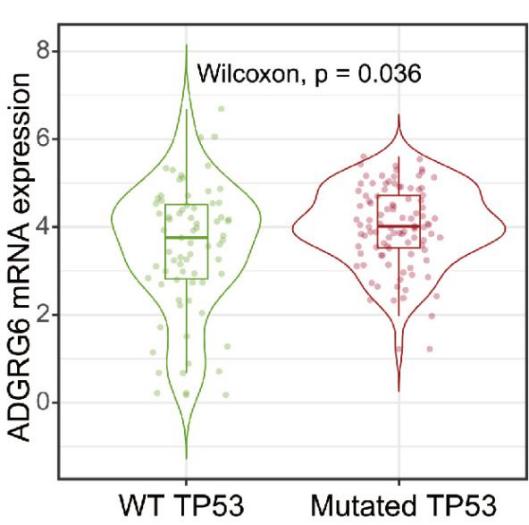
**

**Supplementary Figure S6 | ADGRG6 mRNA expression levels in mutant/wild-type p53 PAAD samples in the TCGA database.**

**Supplementary Tables**

**Supplementary Table S1 | Overall survival (OS)**

**
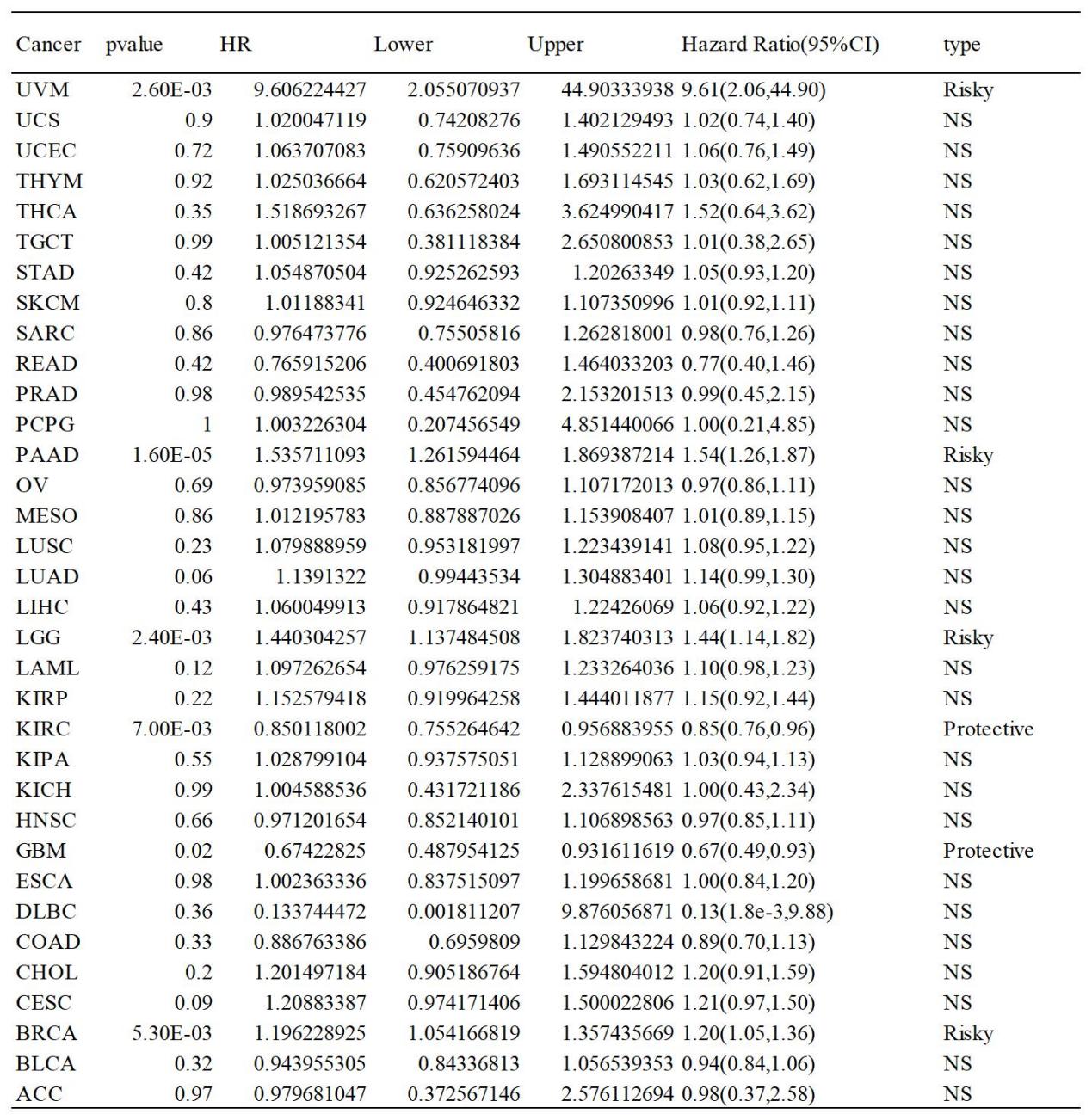
**

**Supplementary Table S2 | Progression-free interval (PFI)**

**
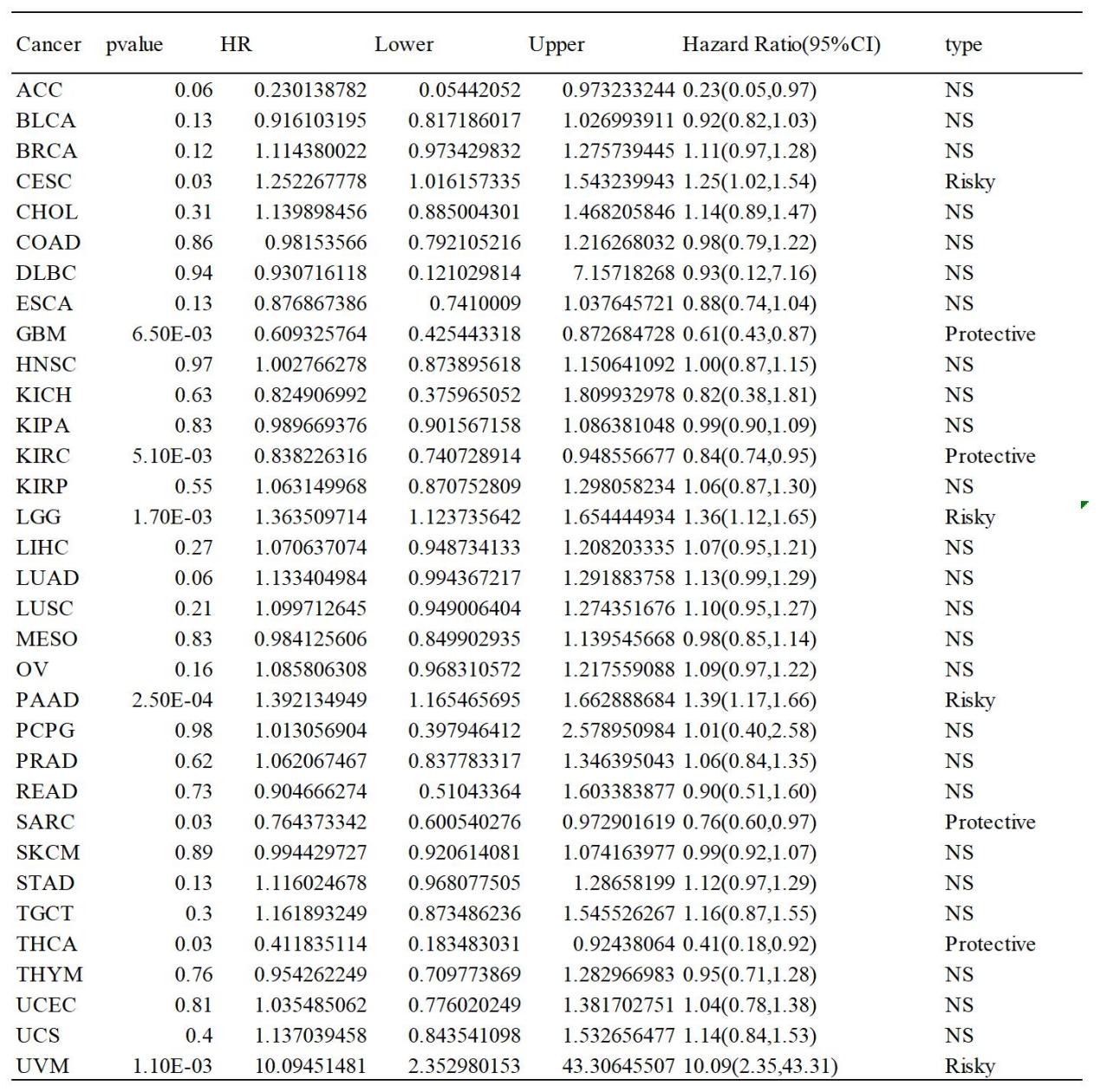
**

**Supplementary Table S3 | Disease-specific survival (DSS)**

**
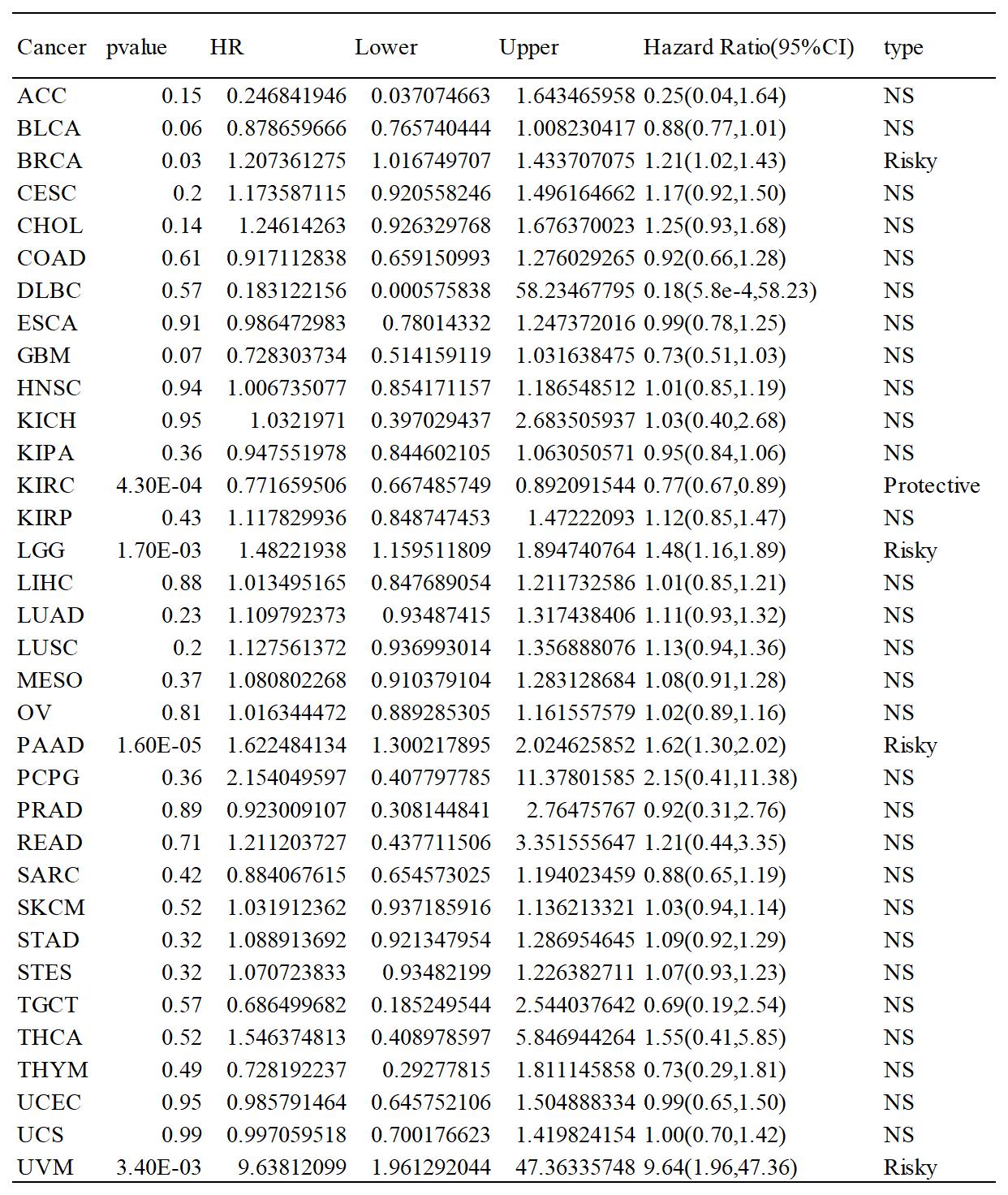
**

**Supplementary Table S4 | Disease-free interval (DFI)**

**
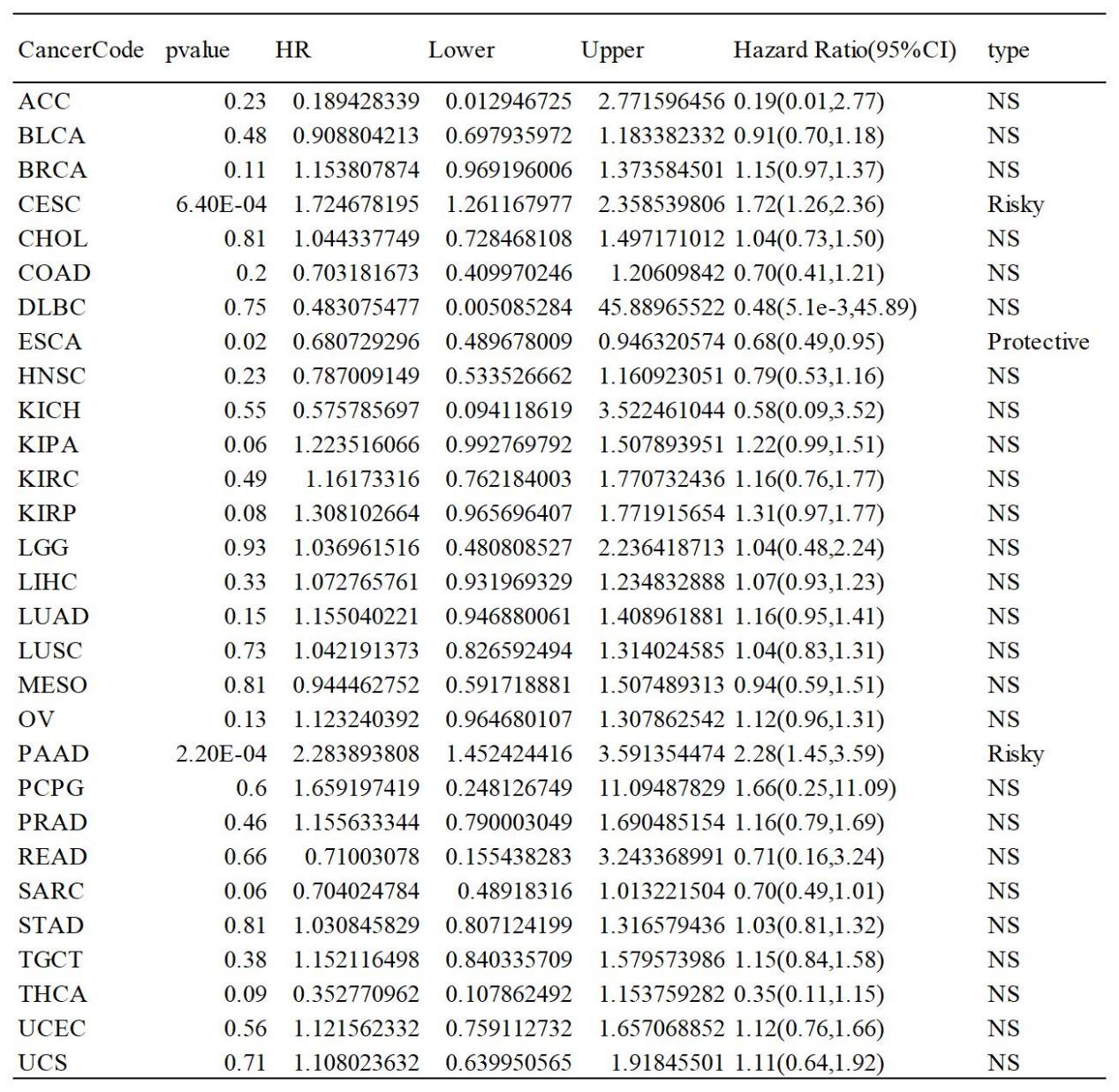
**
